# Harmonization service and global library of models to support country-driven global information on salt-affected soils

**DOI:** 10.1038/s41598-023-40078-9

**Published:** 2023-08-12

**Authors:** C. T. Omuto, M. Scherstjanoi, M. A. Kader, B. Musana, A. Barman, M. Fantappiè, L. S. Jiménez, W. A. Jimenez, H. Figueredo, R. Balta, K. Santander, A. Malatji, A. Nahar, A. Kairat, H. Ahmadzai, J. Morisson, S. Stone, R. Roopnarine, G. Eudoxie, P. Khat, C. Phy, V. Seng, N. Janjirawuttikul, M. Tina, M. Farradas, M. Alferihat, K. Desire, O. J. Jayeoba, M. Loum, W. Ahmad, A. S. Al Rasbi, N. Matolo

**Affiliations:** 1 University of Nairobi, Nairobi, Kenya; 2 Thünen Institute of Forest Ecosystems, Eberswalde, Germany; 3 School of Agriculture, Geography, Environment, Ocean and Natural Sciences, University of the South Pacific, Apia, Samoa; 4 Rwanda Water Resources Board, Kigali, Rwanda; 5 Division of Soil and Crop Management, ICAR-Central Soil Salinity Research Institute, Karnal, Haryana 132001 India; 6 Consiglio per la Ricerca in Agricoltura e l’Analisi dell’Economia Agraria, Centro Agricoltura e Ambiente, Via di Lanciola 12/A, Firenze, Italy; 7 Facultad de Ciencias Exactas y Naturales, Universidad Técnica Particular de Loja, Loja, Ecuador; 8 Dirección de Generación de Geoinformación Agropecuaria, Ministerio de Agricultura y Ganadería, Quito, Ecuador; 9 Ministry of Environment and Water, La Paz, Bolivia; 10 Dirección General de Asuntos Ambientales Agrarios, Ministerio de Desarrollo Agrario y Riego, Huaraz, Peru; 11 Department of Agriculture, Land Reform and Rural Development, Land and Soil Management, Pretoria, South Africa; 12 Soil Resource Development Institute, Ministry of Agriculture, Dhaka, Bangladesh; 13 Al-Farabi Kazakh National University, Almaty, Kazakhstan; 14 Soil Research Directorate, Ministry of Agriculture Irrigation and Livestock, Agriculture Research Institute of Afghanistan, Kabul, Afghanistan; 15 Agricultural Land Management Division, Ministry of Industry Commerce Agriculture and Fisheries, Spanish Town, Jamaica; 16 Faculty of Food and Agriculture, University of the West Indies, St. Augustine Campus, Sangre Grande, Trinidad; 17 Department of Agricultural Land Resources Management, General Directorate of Agriculture, Ministry of Agriculture, Forestry and Fisheries, Phnom Penh, Cambodia; 18 Land Development Department, Ministry of Agriculture and Cooperatives, Bangkok, Thailand; 19 National Agricultural Research Institute, Lae, Papua New Guinea; 20 Ministry of Agriculture, La Habana, Cuba; 21 Soil Survey and Landuse Division, Ministry of Agriculture/Land and Irrigation, Amman, Jordan; 22 Bureau National des Sols (BUNASOL), Ouagadougou, Burkina Faso; 23 Faculty of Agriculture, Nasarawa State University, Keffi, Nigeria; 24 Institut National de Pédologie, Ministère de l’Agriculture et de l’Equipement Rural, Dakar, Senegal; 25 School of Agriculture and Food Sustainability, The University of Queensland, Brisbane, QLD Australia; 26 Ministry of Agriculture, Muscat, Oman; 27 Kenya Agricultural and Livestock Research Organization (KALRO), Nairobi, Kenya

**Keywords:** Ecology, Environmental sciences, Solid Earth sciences

## Abstract

Global distribution of salt-affected soils (SAS) has remained at about 1 billion hectares in the literature over the years despite changes in climate, sea levels, and land use patterns which influence the distribution. Lack of periodic update of input soil data, data gaps, and inconsistency are part of the reasons for constant SAS distribution in the literature. This paper proposes harmonization as a suitable alternative for managing inconsistent data and minimizing data gaps. It developed a new harmonization service for supporting country-driven global SAS information update. The service contains a global library of harmonization models for harmonizing inconsistent soil data. It also contains models for identifying gaps in SAS database and for showing global distribution where harmonization of available data is needed. The service can be used by countries to develop national SAS information and update global SAS distribution. Its data availability index is useful in identifying countries without SAS data in the global database, which is a convenient way to identify countries to mobilize when updating global SAS information. Its application in 27 countries showed that the countries have more SAS data than they currently share with the global databases and that most of their data require SAS harmonization.

## Introduction

Global distribution of salt-affected soils (SAS), which is influenced by climate, soil parent material, proximity to salty water, and land use, was expected to have changed in the past decades because of changes in global climate, sea levels, land use patterns, and agricultural intensification and modernization. However, the distribution is still portrayed in the literature at about one billion hectares since 1980s^1–5^. This may be partly due to some challenges with the input data for SAS mapping, which are mostly global soil maps^6,7^, expert opinions^5^, remote sensing images and climate maps^3^, and soil databases^2,4^. Updating these input data has had many challenges such as lack of sustained coordination and mobilization of data holders, data inconsistencies, copyright, and data gaps^8^. Recently, the Global Soil Partnership (GSP) of the Food and Agriculture Organization of the United Nations (FAO) attempted to overcome the challenge of coordination and mobilization of soil data holders by use of a country-driven approach^9^. In this approach, representatives from the countries are mobilized and their technical capacity strengthened towards the development of their national SAS information database. The national databases are then contributed to the global SAS information database. While this approach presents a viable alternative for more data and less gaps in global SAS database, it still has limitations due to inconsistencies that are typical of crowdsourced data. Appropriate harmonization is one way of overcoming some of these challenges. This paper developed a framework for harmonizing crowdsourced soil data to support harmonized global SAS information.

Most recent global maps of SAS have been produced using publicly available soil databases^2–4^ from more than 90 countries^10–12^. Although the databases have opened new opportunities for mapping global SAS using measured soil properties, they still have gaps. Many countries are not represented in the databases while some countries are only represented with data that were collected in the late 20^th^ Century. In addition to data gaps, the databases also have typical discordance in crowdsourced data such as inconsistent sampling dates, sampled depths, and measurement methods for soil properties. Impact of the discordance is evident in the way that some SAS estimation methods discard non-conforming parts of the database, which further creates more data gaps and potential for high uncertainties in the final SAS information. Appropriate harmonization is proposed to partially overcome some of the inconsistencies in SAS data and substantially reduce the number of data points which would otherwise be omitted during SAS information development.

Popularly used soil properties for SAS assessment that are often targeted for harmonization are electrical conductivity (EC), pH, exchangeable sodium percent (ESP), sodium adsorption ratio (SAR), total soluble salts (TSS), and total dissolved solids (TDS)^13,14^. Most SAS classification schemes using these soil properties recommend measurements taken in extract solutions from saturated soil paste as the standard^15–17^. Harmonization aims to convert values obtained from other extracts or methods to the equivalent values of the extract from saturated soil paste. Most conversion models in the literature were developed without the focus for improving global SAS information and therefore were not adequately tested in large soil databases to evaluate their rigor at the global level. In this paper, robust conversion models were developed and tested on the global databases using mixed-effects modelling approach^18^. Mixed-effects models are suitable for modelling data with more than one source of random variability or where measurements are clustered. They have potential application in modelling soil characteristics that are influenced by natural groups such as texture^19^. Presently, most conversion models for SAS soil properties recognize the influence of these soil groups on the conversion models but do not necessarily integrate them in the modelling process^20^. In this paper, these soil groups were incorporated in the mixed-effects harmonization models to improve models’ accuracy and robustness. The goal of this paper was to show how a harmonization service based on the mixed-effects harmonization modelling and open-source software package can support harmonized global SAS information.

## Results

### Harmonization models

**Figure 1:**
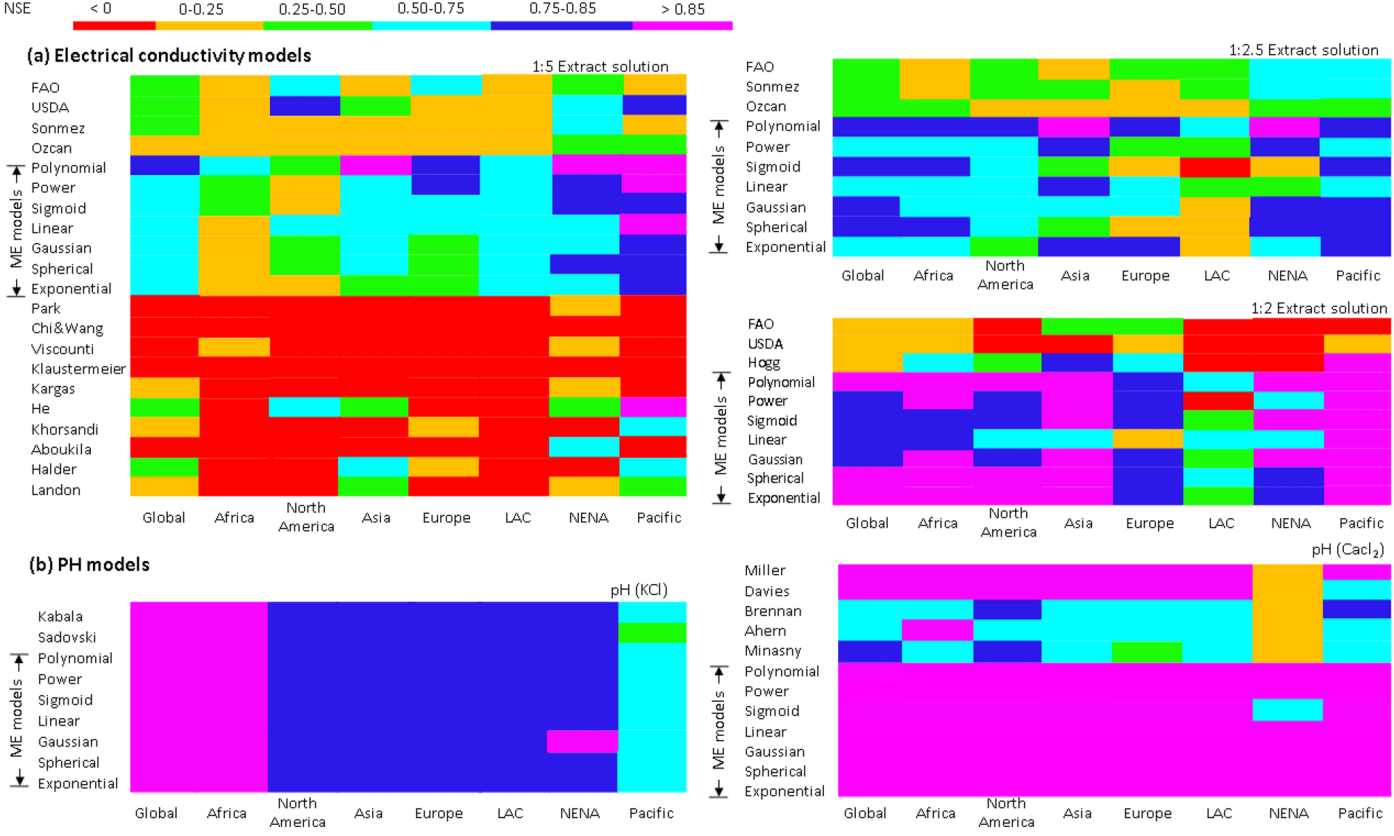
Performance evaluation for EC and pH models on validation data grouped according to geographic regions (LAC—Latin America & Caribbean, NENA—Near East and North Africa).

A new SAS harmonization service was developed and contains a global library of models for harmonizing SAS data. The library has 37 models for EC and pH harmonization. Evaluation of these models using the global datasets showed that mixed-effects (ME) models produced the best harmonization of soil EC and pH. They had the highest predictive statistics on the validation dataset (r^2^ > 0.7, Nash–Sutcliffe coefficient of efficiency (NSE) > 0.5, Root Mean Square Error (RMSE) < 1). Not only did they perform very well globally but also in soil data from most regions of the world. This is shown in Fig. 1 where the models were tested on the validation dataset from different geographic regions. ME models comprise fixed effects which are overall average model parameters and random effects which are random variations around the fixed effects. Random effects can be further modelled with factors which are believed to influence variations of the model parameters such as soil texture and consequently improving ME predictive performance. When the random effects were modelled with soil textural classes performance of ME models significantly improved (due to more than 7% reduction in residual standard errors (RSE) and more than 1.2% increase in r^2^ and NSE) (supplementary Fig. S4.4). Low RSE and high r^2^ and NSE are diagnostic indicators of better predictive performance of models^21,22^. More improvements in ME models’ performance (due to 10% decline in RSE and 1.5% increase in r^2^ and NSE) were also obtained when regional data categorization was included in the random-effects modelling (supplementary Fig. S4.5). Regional data categorization grouped the global data into geographic regions such as sub-Saharan Africa, Asia, Europe, Latin America and the Caribbean (LAC), Near East and North Africa (NENA), North America, and the Pacific (supplementary Fig. S1.5). Improvements in ME models with inclusion of soil texture and regional groups implied that natural soil groups are important in the harmonization of EC and pH.

**Figure 2:**
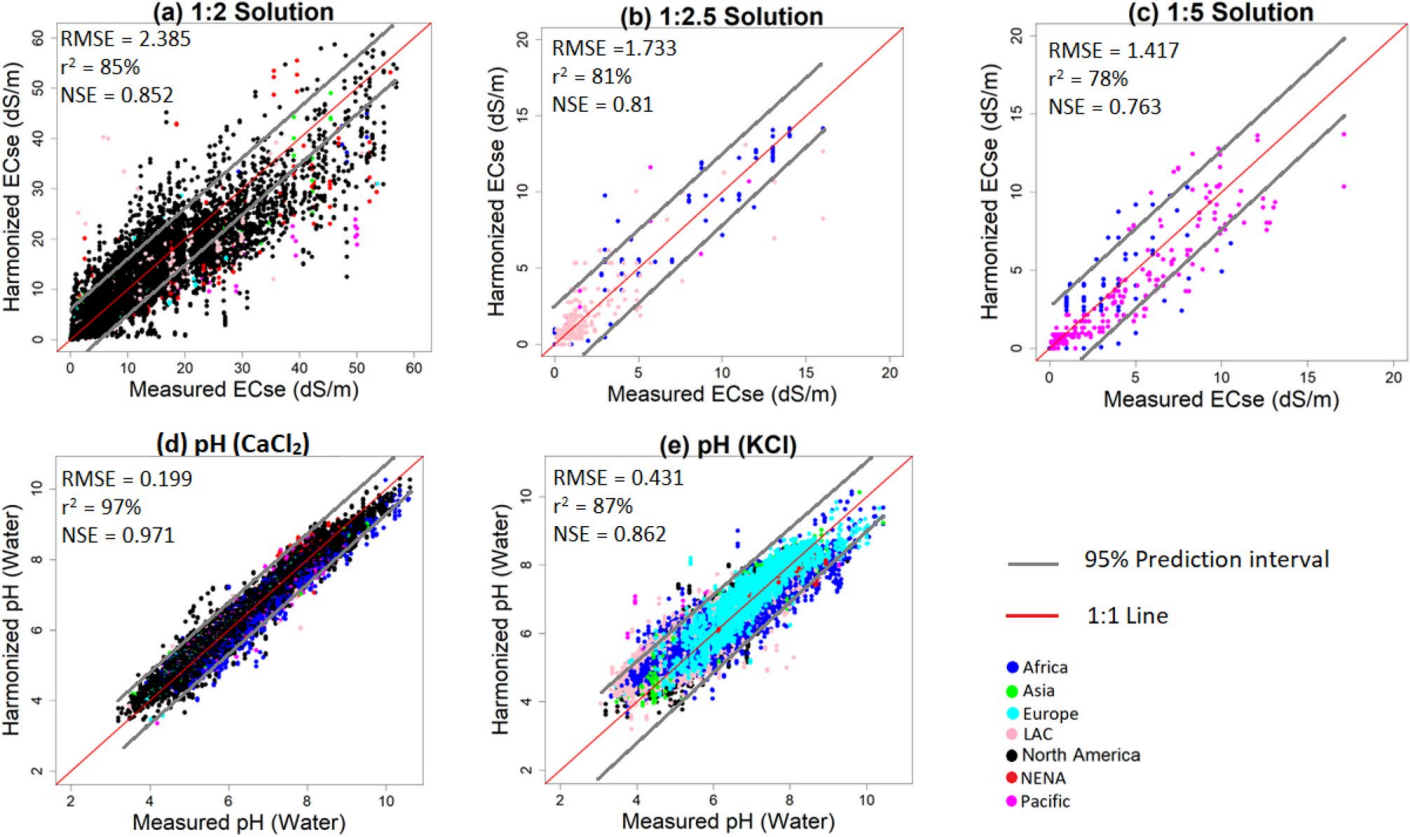
Comparison of ME polynomial harmonized and measured ECse and pH(water) using holdout samples.

Further evaluation of the harmonization models was done to assess their performance in high (EC ≥ 8 dS/m and pH ≥ 7) and low value ranges (EC < 8 dS/m and pH < 7). The results showed that the models with simple linear relationships exhibited very low performance for high EC values (NSE < 0.2). Most models from the literature were of the form of a simple linear relationship (Supplementary Table S4.1). Their low performance for high EC values implies that they are less certain in identifying high intensity SAS classes. ME models showed the best performance for all EC ranges. This was depicted in graphical summaries where they portrayed relatively spread-out prediction throughout the range of measured values (Fig. 2). Therefore, they can identify all SAS intensity classes better than most models in the literature.

High EC data range (EC ≥ 8 dS/m) had high variability and were fewer than low EC data (EC < 8 dS/m) (supplementary Fig. S2.1a). They were mostly from North America, NENA, and Latin America and were modelled with high uncertainty due to their characteristic variability. More calibration data are recommended to improve their harmonization. Assessment of standardized residual plots showed that some of the high EC data that came from the United States of America (USA), Oman, United Arab Emirates (UAE), Russia, central Asia, Antarctica, Puerto Rico, and Chile appeared as outliers (supplementary Fig. S4.3). Most of these data points came from areas which did not have adequate representation of SAS data in the global soil databases (supplementary material S1). More calibration data were also recommended to improve performance of the harmonization models in these areas.

### Harmonization service with global library of harmonization models

SAS harmonization service is focused on harmonizing soil EC and pH and on provision of information on available global SAS data. Its harmonization application facilitates consistent SAS intensity classification using three categories of models: models based on the mixed effects (ME) approach, models developed from existing expressions in the literature, and generic expressions for users to customize their own harmonization models. Generic models for developing own harmonization models are available for three different harmonization scenarios: (1) where ECse or pH(water) and corresponding non-standard EC or pH are available for a subset of SAS database; (2) where a relationship is needed between ECse or pH(water) and in-situ measurements from bulk soil sensors (such as electromagnetic induction); and (3) where a relationship is needed between ECse or pH(water) and other soil properties. In all these scenarios, the service can be used to develop own harmonization models on a subset of the data and then applying the models on the remainder larger part of the SAS database.

**Figure 3:**
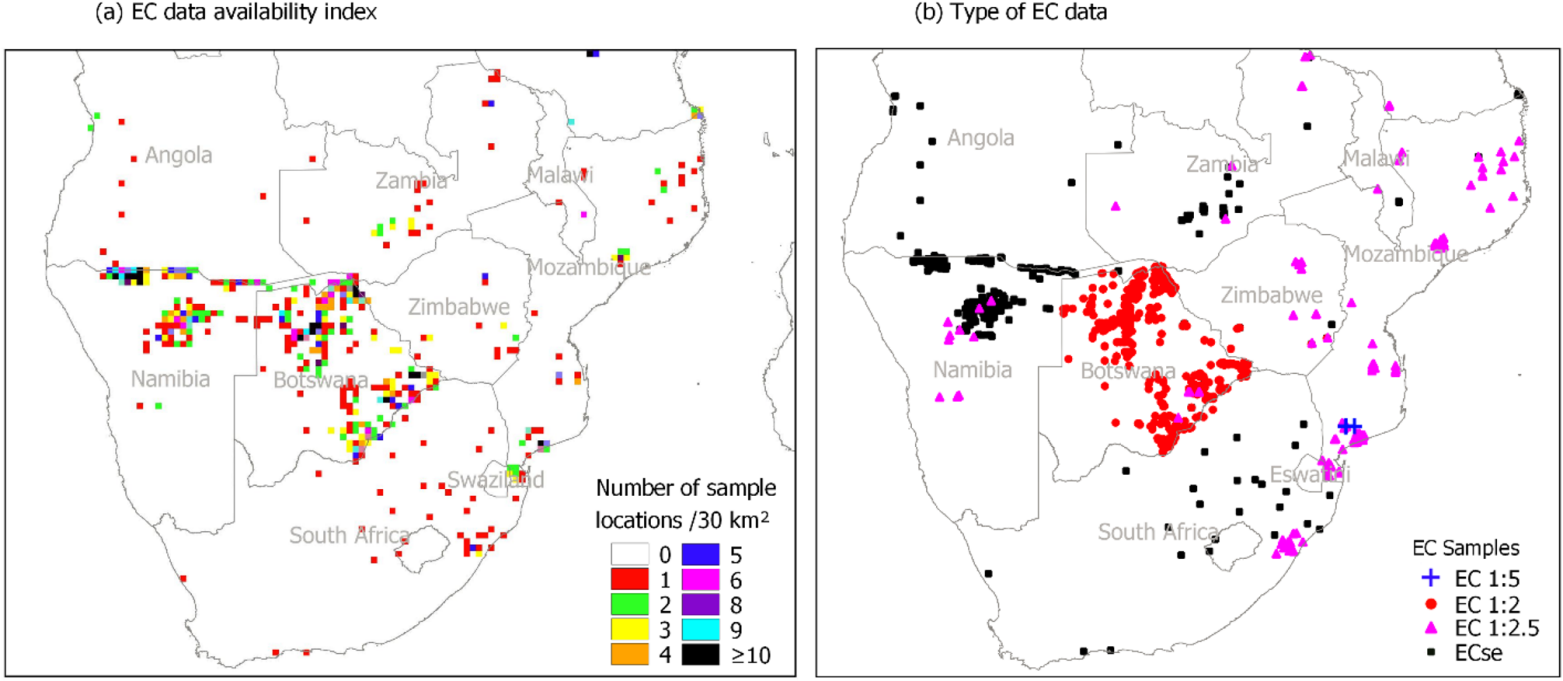
Example service application on global SAS data availability in southern Africa.

In addition to harmonization, the service also provides information on global SAS data availability and predictive performance of various harmonization models in different parts of the world (Fig. 1). Its data availability index is for spatial visualization of global SAS data availability. The index is useful in identifying spatial gaps in SAS data. Example application of the index in Fig. 3a demonstrates how it identifies available SAS data at a spatial resolution of 30 km^2^. It depicts most southwestern parts of the region without EC data in the global SAS database. The identified gaps can then be targeted with input data mobilization to update the global SAS database. In another perspective, comparison of the index with the map of types of SAS data illustrates areas where available data need harmonization. In Fig. 3b, these areas are shown in Botswana, Zambia, and Mozambique where there are many locations without the standard data for SAS intensity classification. If non-standard data are removed when developing SAS intensity classification for this region, more data gaps will be created. Harmonization of non-standard data is an alternative way to reduce these gaps. In this regard, the data availability index can be used to identify non-standard SAS data in the global database to target with harmonization.

**Figure 4:**
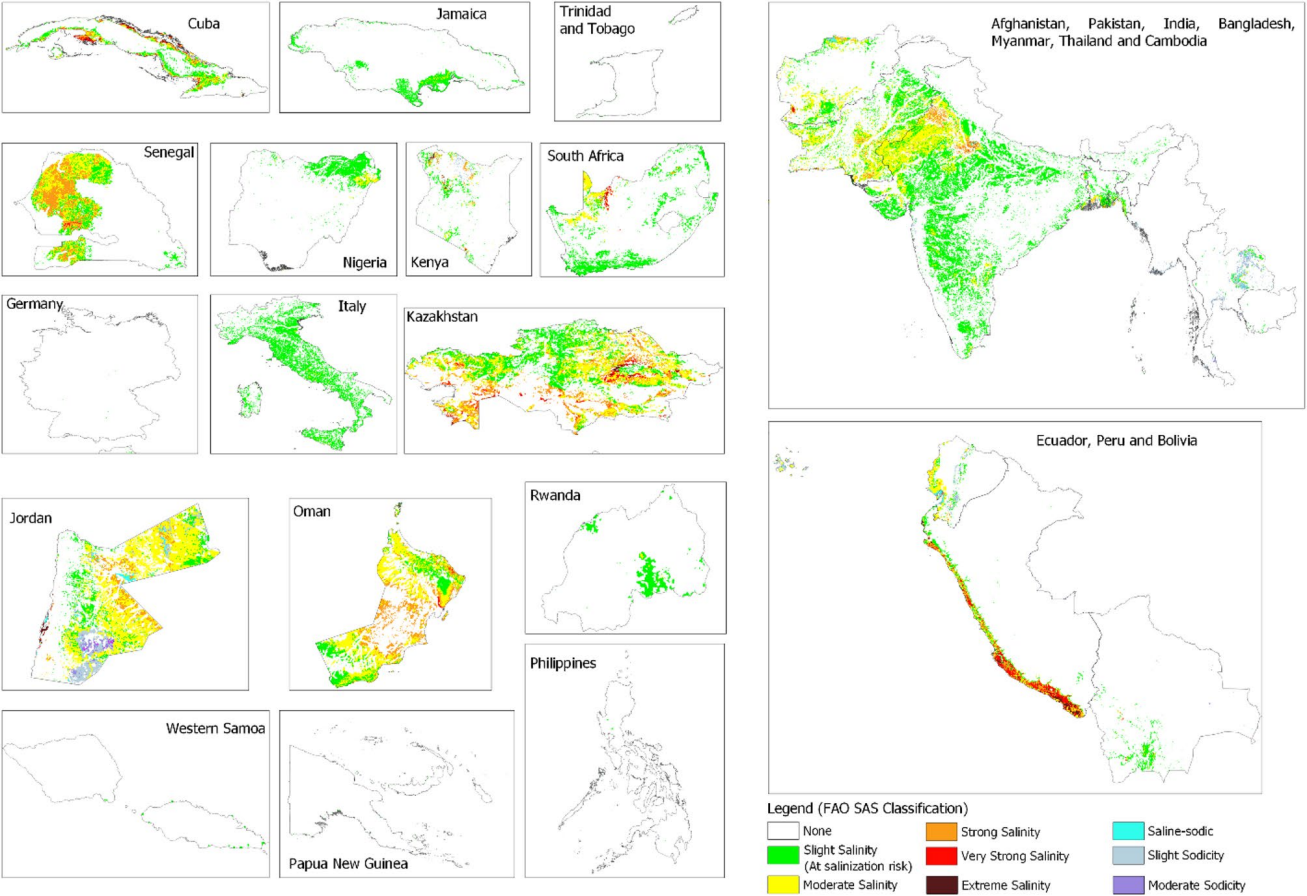
Long-term average topsoil (0–30 cm) SAS intensity classes for case-study countries based on FAO classification^14,15^.

Application of the harmonization service in 27 countries showed that there were more SAS data in most countries that have not been shared with the global datasets. On average, the data availability index was more than 100 times higher in the case-study countries than the corresponding global data availability index within these countries. Less than half of the case-study countries had ECse and pH (water), which implies that most countries needed harmonization service to improve their SAS information. Therefore, the country-level data were first harmonized before developing national SAS intensity classes. The resultant SAS intensity classification showed predominance of coastal salinity in most countries from Latin America, the Caribbean, Pacific Islands, and southeast Asia countries (Fig. 4 and supplementary Table S6.2). It also showed that strong and very strong salinity are dominant in arid areas while sodic soils were identified in few locations in northern Kenya, Jordan, and Thailand. Areas with these soils have been associated with natric mineralogy of the underlying parent rocks^23–25^. The case study application established that the choice of harmonization model influenced the accuracy and spatial distribution of the resultant national SAS intensity classes. Harmonization models with poor prediction of high EC and pH values produced high misclassification of SAS intensity classes (supplementary Fig. S6.2). Information from the harmonization service guided selection of the best regional models to harmonize country-level data.

## Discussions

One of the challenges in building global soil information from crowdsourcing is how to deal with inconsistencies in input soil data^26^. Harmonization provides a partial solution to this challenge^27^. Presently, there is no clear collection of a suite of harmonization models to support consistent global SAS information development. In addition, most available models have not been adequately tested on the global datasets to assess their performance. The global library of harmonization models in this study presents convenient access to over 30 different models in one collection. It opens the door for comparing and testing of models, development of new harmonization models, and for improving SAS information development. This study used a model testing approach which is useful in guiding selection of proper harmonization model from the library. The approach targets different ranges of measured EC and pH in various geographic regions to identify harmonization performance in low and high SAS intensity classes in these regions (Fig. 1 and supplementary material S4). This is necessary to minimize misclassification of SAS intensity classes and subsequent misrepresentation of SAS information. For example, FAO, Ozcan, and USDA models^15,17,28^ were shown with relatively poor harmonization of EC in Africa (Fig. 1) and low prediction of high EC values (supplementary Fig. S4.1). Their use in predicting SAS intensity class produced high misclassification and misrepresentation of SAS classes in northern Kenya (supplementary Fig. S6.2).

The harmonization approach and global library of harmonization models demonstrated an alternative way of incorporating useful SAS input data which otherwise would be left out when developing SAS information. More data gaps are inevitable without harmonization of available non-standard soil data. For example, in Fig. 3, most data points from eastern Botswana and the whole of Mozambique would be removed if no harmonization is used. Perhaps this is one of the contributing factors for low SAS prediction in these areas in the literature where global datasets have been used and non-standard data excluded from the analyses^3,4^. By reducing the data gaps, the harmonization service facilitates reduction of uncertainties due to gaps in spatial SAS information. Besides facilitating reduction of data gaps, the library of models can also be used to supports selection of appropriate harmonization model to further minimize uncertainty in SAS information development. For example, models with low predictive performance for high EC and pH (supplementary Fig. S4.1) may not be preferred in areas dominated with high SAS intensities because they are likely to produce high uncertainties (supplementary Figs. S6.2 and S6.3). The library offers alternative models with better performance to improve SAS information development in such areas. In general, the service can be used to identify possible sources of uncertainties in spatial SAS information (for example, due to data gaps or inadequate harmonization model). It also offers alternative ways for partially overcoming the uncertainties such as through recommendations for more representative samples in areas with data gaps and use of better harmonization models.

The harmonization service provides a platform for improving contribution to and use of global SAS information. Its data availability index shows areas where input data are currently available in the global datasets. SAS data users can use it to query data availability in any area of interest. The index can also be used to mobilize input data contribution to the global data where there are gaps in the global datasets. For example, currently the index shows no EC data for Myanmar and Cambodia in the global datasets (supplementary Fig. S1.1). However, this study has shown that there are SAS data in these countries (Supplementary Table S6.1). Cases such as in Myanmar and Cambodia can be encouraged to develop their SAS information and contribute to the global SAS information. In this regard, the service can be used to support country-driven updates of global SAS information^9^. Since most countries have more national data density than is represented in the global datasets, the countries can use the service to develop national SAS information and contribute the output to global SAS information (Fig. 4).

## Conclusions

A new harmonization service was developed for supporting SAS information development. It contains a global library of harmonization models for harmonizing soil data, which can be used to improve consistent global SAS information. Consistent input soil data are a major challenge in global mapping of salt-affected soils. The service also contains models for querying availability of global SAS datasets and for guiding further actions to improve data gaps. Not only does it identify data gaps but also shows where data harmonization is needed. Evaluation of the harmonization models showed differences in performance for high or low EC and pH values in different regions of the world. Models based on the mixed-effects (ME) approach were found to be adequate in harmonizing low and high EC and pH values in most parts of the world. ME approach can also be used to modify existing harmonization models in the literature to improve their predictive performance.

The harmonization service has a provision for developing models which target other SAS data types that were not tested in this study. Harmonization models for these types of data and their evaluation is recommended. Data availability index in the service is recommended as a tool to facilitate SAS data mobilization for countries without SAS data.

## Methods

### Input data for developing global harmonization models

This study used EC, pH, and texture data from the global soil databases^10–12^. The databases contained four types of EC data depending on the soil extract solution used in the EC measurement^29^. They include EC in 1:2 extract solution (denoted as EC_1:2_ in this study), EC in 1:2.5 extract solution (EC_1:2.5_), EC in 1:5 extract solution (EC_1:5_) and EC in saturated soil paste extract (ECse). The data had 138,530 EC_1:2_ samples; 148,176 EC_1:2.5_ samples; 167,015 EC_1:5_ samples; and 194,119 ECse samples. Spatial distribution of the data is given in supplementary Fig. S1.1. In addition, the data also contained 127,491 samples with EC_1:2_ and ECse; 2,981 samples with EC_1:2.5_ and ECse; and 1,566 samples with EC_1:5_ and ECse. All samples had measurements of soil particle size distribution (% sand, % silt, and % clay contents). Since the databases already harmonized the limits between particle sizes, no further harmonization was done on the textural proportions between samples. The data also contained 228,129 soil samples with pH (water), 214,383 samples with pH (KCl), 244,270 samples with pH (CaCl_2_), 228,129 samples with both measurements of pH(water) and pH (CaCl_2_), and 198,135 samples with both measurements of pH(water) and pH (KCl). Details of the methods for measuring the soil properties are given in the database documentation^10–12^.

### Harmonization models based on mixed-effects approach

Mixed-effects harmonization models were developed from the general relationship in Eq. (1) between harmonized (*y*) and measured (*x*) soil properties.

1 $$ y_{i} = f\left( {x_{i} ,\theta } \right) + g\left( {\varepsilon_{i} } \right)\quad {\text{for 1}} \le i \le n $$

where *f* is the harmonization function, *θ* is a set of model-fitting parameters, *ε* is the residual representing the difference between *x* and *y*, *g* represents the function for the residuals, and *n* is the number of observations. Although most models in the literature recognize that harmonization varies with soil textural classes, soil types, (and other factors), they rarely incorporate these factors in their structures other than providing different models for different factor groups^15,30^. Equation (1) was modified as mixed-effects model to incorporate factor-groups in the harmonization such as the textural classes (Eq. 2). Significant differences were found in SAS soil properties between soil textural classes (Supplementary Fig. S1.4) that could be modelled using mixed-effects models.

2 $$ \begin{gathered} y_{ij} = f\left( {x_{ij} ,\varphi_{i} } \right) + g\left( {\varepsilon_{ij} } \right)\quad \,for{1} \le i \le \, n \hfill \\ \varphi_{j} = \theta + b_{j} \quad \quad \quad \quad \quad \quad \quad {\text{for 1}} \le j \le m \hfill \\ \end{gathered} $$

where *b* is a set of random-effects for *m* groups (e.g., textural classes), and *θ* represents model-fitting parameters comprising global average parameters (also known as fixed-effects). The model description and computation are given in the supplementary material S2. Random-effects account for between-groups variability and complements within-groups random residual term in improving the model’s performance^18,19^. Equation (2) was used to harmonize EC and pH.

Seven different models for *f* function in Eq. (2) were chosen based on the orientation of the scatterplot between EC in saturated paste extract (ECse) and EC in other soil extracts and the scatterplot between pH (water) and pH (KCl) or pH (CaCl_2_) (supplementary Fig. S2.1). The models and their curve-fitting parameters are summarized in the supplementary Table S2.1.

### Performance evaluation

Predictive performance of the harmonization models was evaluated by comparing harmonized values with measured ECse or pH (water) values. To test the predictive performance, the global dataset was stratified by soil textural classes and randomly split into two parts: one part for developing the harmonization models and the other part held out for evaluating model performance (see supplementary material S3). Predictive performance was evaluated using models’ accuracy and uncertainty. Statistical indices for evaluating model accuracy were correlation (r^2^), Nash–Sutcliffe coefficient of efficiency (NSE), root-mean square error (RMSE), and bias. Models with lowest RMSE and bias and highest r^2^ and NSE were considered as best. Visual inspection of graphical plots of harmonized versus measured soil properties^31^ was also done to show if the model had balanced prediction throughout the range of measured values. Uncertainty was evaluated using models’ prediction interval at 95% confidence interval^32^. Models with narrow intervals and most data points within interval limits were considered more certain than those with wide intervals and most data points were outside the interval limits.

Performance evaluation indices were compared for three different data scenarios to assess models’ robustness: (1) whole range of measured values (between minimum and maximum values); (2) low data values (e.g., EC < 8 dS/m and pH < 7); and (3) high values (e.g., EC ≥ 8 dS/m and pH ≥ 7). A harmonization model was considered robust if it had the best evaluation statistics for these three scenarios. Analysis of low and high values also facilitated identification of model areas with poor predictive performance so that they can be targeted with more input data to improve the models.

### Harmonization service

A harmonization service for SAS information was developed. The service has two main application areas: EC and pH harmonization and information provision on available global SAS data. Its harmonization application contains mixed effects (ME) models, existing models from the literature, and generic expressions to aid customization of own harmonization model. The generic expression of the form of Eq. (1) accommodates prediction of ECse or pH (water) from a variety of independent variables such as EC from bulk soil sensors (e.g., electromagnetic induction), EC from soil extracts other than those shown in this study, and pedo-transfer functions with other soil properties. An open-source computer codes for implementing these models was developed using R software^33^ (supplementary material S5).

Information on available SAS data was based on the global distribution of sample locations. The index in Eq. (3) was developed to assess SAS data availability.

3 $$ data\;availaibility\;index = \frac{samples}{{area}} $$

where *samples* are number of sample locations with distinct latitude and longitude coordinates and *area* is a spatial square grid (resolution) containing the samples. Dimensions of the square grid is optionally selected from values such 1, 5, 10, 20 km^2^, etc. for an area of interest.

The harmonization service was tested in 27 countries to facilitate national SAS information development (supplementary material S6). This test application illustrated how the service can be used to support harmonized national SAS information development.

### Supplementary Information

Supplementary Information.

## Data Availability

The global datasets generated during and/or analyzed during the current study are available in the ISRIC Data Hub (https://data.isric.org/geonetwork/srv/eng/catalog.search#/home), LUCAS Topsoil Data (https://esdac.jrc.ec.europa.eu/resource-type/datasets), and FAO Soil Portal for HWSD (https://www.fao.org/soils-portal/data-hub/soil-maps-and-databases/harmonized-world-soil-database-v12/en/). Case study country data are available from the authors upon reasonable request.
