# Supplementary material for: Harmonization service and global library of models to support country-driven global information on salt-affected soils
Source: Sci Rep. 2023 Aug 12;13:13157. doi: 10.1038/s41598-023-40078-9 (PMC10423227; doi:10.1038/s41598-023-40078-9)
Supplement: Supplementary file 1 — Supplementary Information. [file 41598_2023_40078_MOESM1_ESM.docx]

**Supplementary Information**

**Harmonization service and global library of models to support country-driven global information on salt-affected soils**

Omuto, CT^1*^, Scherstjanoi, M^2^, Kader, M.A^3^, Musana, B^4^, Barman, A^5^, Fantappiè, M^6^, Jiménez, L.S^7^, Jimenez, W.A^8^, Figueredo, H^9^, Balta-Crisologo, R.A^10^, Santander Hidalgo Candia, K.E^10^, Malatji, A^11^, Nahar, A^12^, Kairat, A^13^, Ahmadzai, H^14^, Morisson, J^15^, Stone, S^15^, Roopnarine, R^16^, Eudoxie, G^16^, Phy, C^17^, Khat, P^17^, Seng, V^17^, Janjirawuttikul, N^18^., Tinah, M^19^, Farradas, M^20^, Alferihat, M^21^, Desire, K^22^, Jayeoba, O.J^23^, Loum, M^24^, Ahmad, W^25a,b^, Salim, A^26^, Matolo, N^27^

^1^University of Nairobi, Kenya

^2^Thünen Institute of Forest Ecosystems, Germany

^3^School of Agriculture, Geography, Environment, Ocean & Natural Sciences, University of the South Pacific, Apia, Samoa

^4^Rwanda Water Resources Board, Rwanda

^5^Division of Soil and Crop Management, ICAR-Central Soil Salinity Research Institute, Karnal 132001, Haryana, India

^6^Consiglio per la Ricerca in Agricoltura e l'Analisi dell'Economia Agraria, Centro Agricoltura e Ambiente, via di Lanciola 12/A, Firenze, Italy

^7^Facultad de Ciencias Exactas y Naturales, Universidad Técnica Particular de Loja, Loja, Ecuador

^8^Dirección de Generación de Geoinformación Agropecuaria, Ministerio de Agricultura y Ganadería, Quito, Ecuador

^9^Ministry of Environment and Water, Bolivia

^10^Dirección General de Asuntos Ambientales Agrarios, Ministerio de Desarrollo Agrario y Riego, Peru

^11^Land and Soil Management, Department of Agriculture, Land Reform & Rural Development, Pretoria, South Africa

^12^Soil Resource Development Institute, Ministry of Agriculture, Dhaka, Bangladesh

^13^Al-Farabi Kazakh National University, Almaty, Kazakhstan

^14^Soil Research Directorate, Agriculture Research Institute of Afghanistan, Ministry of Agriculture Irrigation and Livestock, Kabul, Afghanistan

^15^Agricultural Land Management Division, Ministry of Industry Commerce Agriculture & Fisheries, Jamaica

^16^University of the West Indies, Faculty of Food and Agriculture, St. Augustine Campus, Trinidad

^17^Department of Agricultural Land Resources Management, General Directorate of Agriculture; Ministry of Agriculture, Forestry and Fisheries, Cambodia

^18^Land Development Department, Ministry of Agriculture and Cooperatives, Bangkok, Thailand

^19^National Agricultural Research Institute, Papua New Guinea

^20^Directorate of Soils and Fertilizers, Ministry of Agriculture, Cuba

^21^Soil Survey and Landuse Division, Ministry of Agriculture/Land and Irrigation, Jordan

^22^Bureau National des Sols (BUNASOL), Ouagadougou, Burkina Faso

^23^Faculty of Agriculture, Nasarawa State University, Keffi, Nigeria

^24^Institut National de Pédologie, Ministère de l’Agriculture et de l’Equipement Rural, Dakar, Sénégal

^25^School of Agriculture and Food Sustainability, The University of Queensland, St. Lucia 4072, Queensland, Australia

^26^Ministry of Agriculture, Oman

^27^Kenya Agricultural and Livestock Research Organization (KALRO), Kenya

**Table of contents**

[1. Global data for developing and testing SAS harmonization models 3](#_Toc138959212)

[2. Fitting mixed-effects harmonization models 6](#_Toc138959213)

[3. Data splitting for calibrating and validating harmonization models 7](#_Toc138959214)

[4. Performance evaluation of harmonization models 10](#_Toc138959215)

[5. Harmonization service 15](#_Toc138959216)

[6. Application of harmonization service in national SAS information development 17](#_Toc138959217)

[References 25](#_Toc138959218)

#### 1. Global data for developing and testing SAS harmonization models


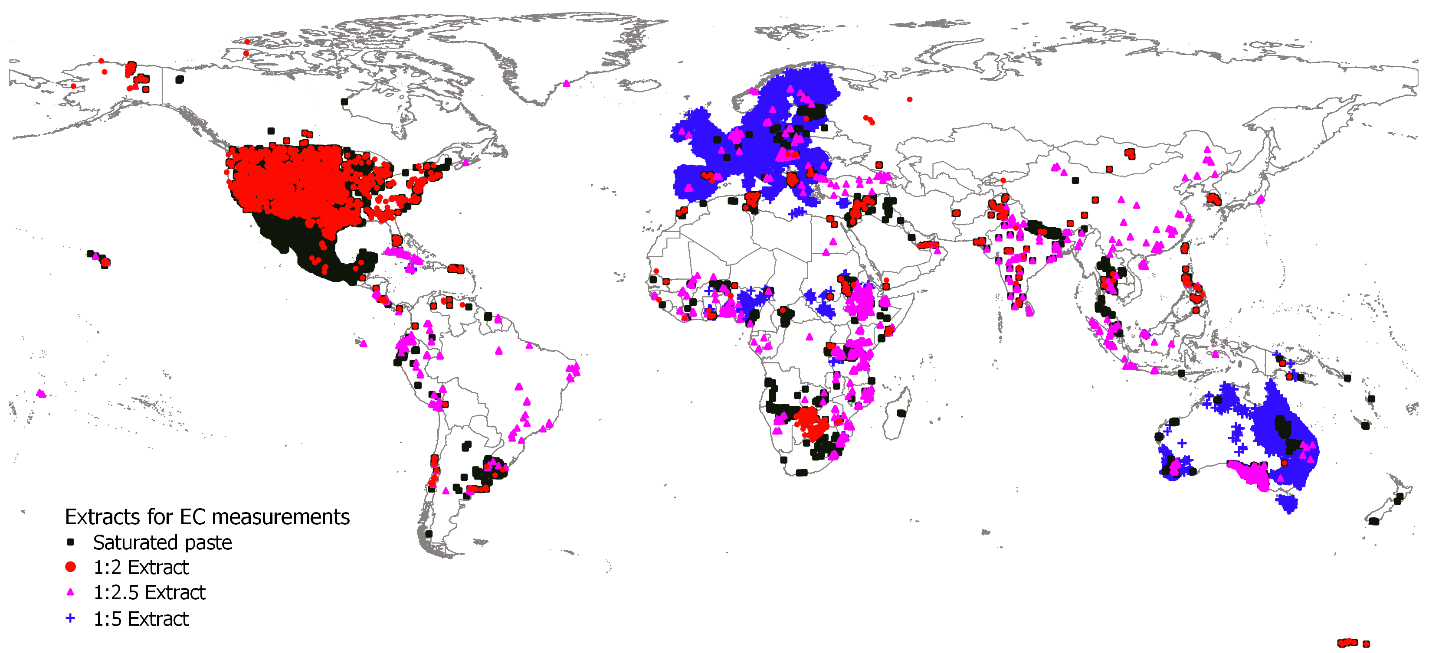


Figure S1.1: Spatial distribution of EC data in global datasets (data source: WoSIS^1^ and LUCAS^2^)


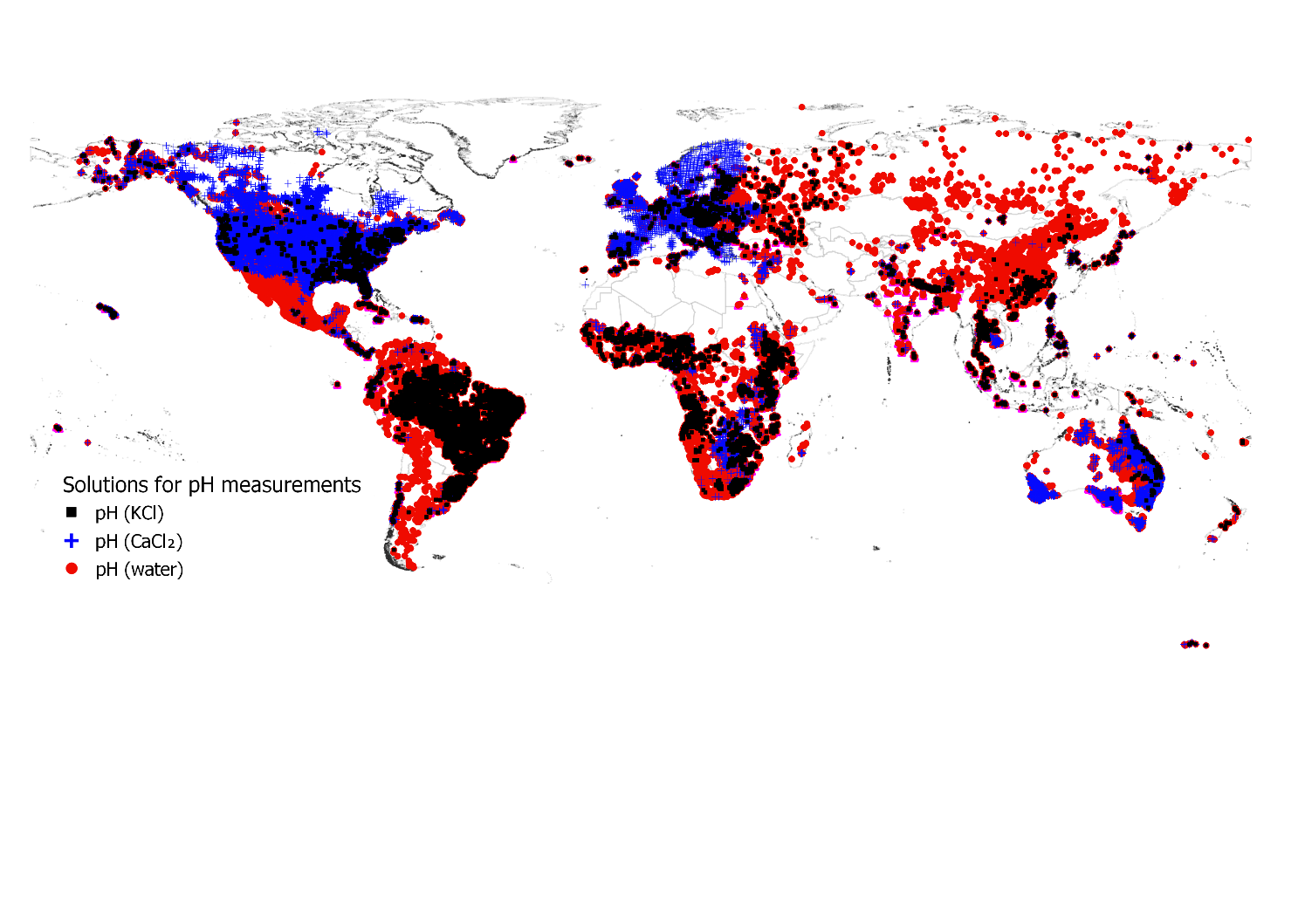


Figure S1.2: Spatial distribution of pH data in global datasets (data source: WoSIS^1^ and LUCAS^2^)


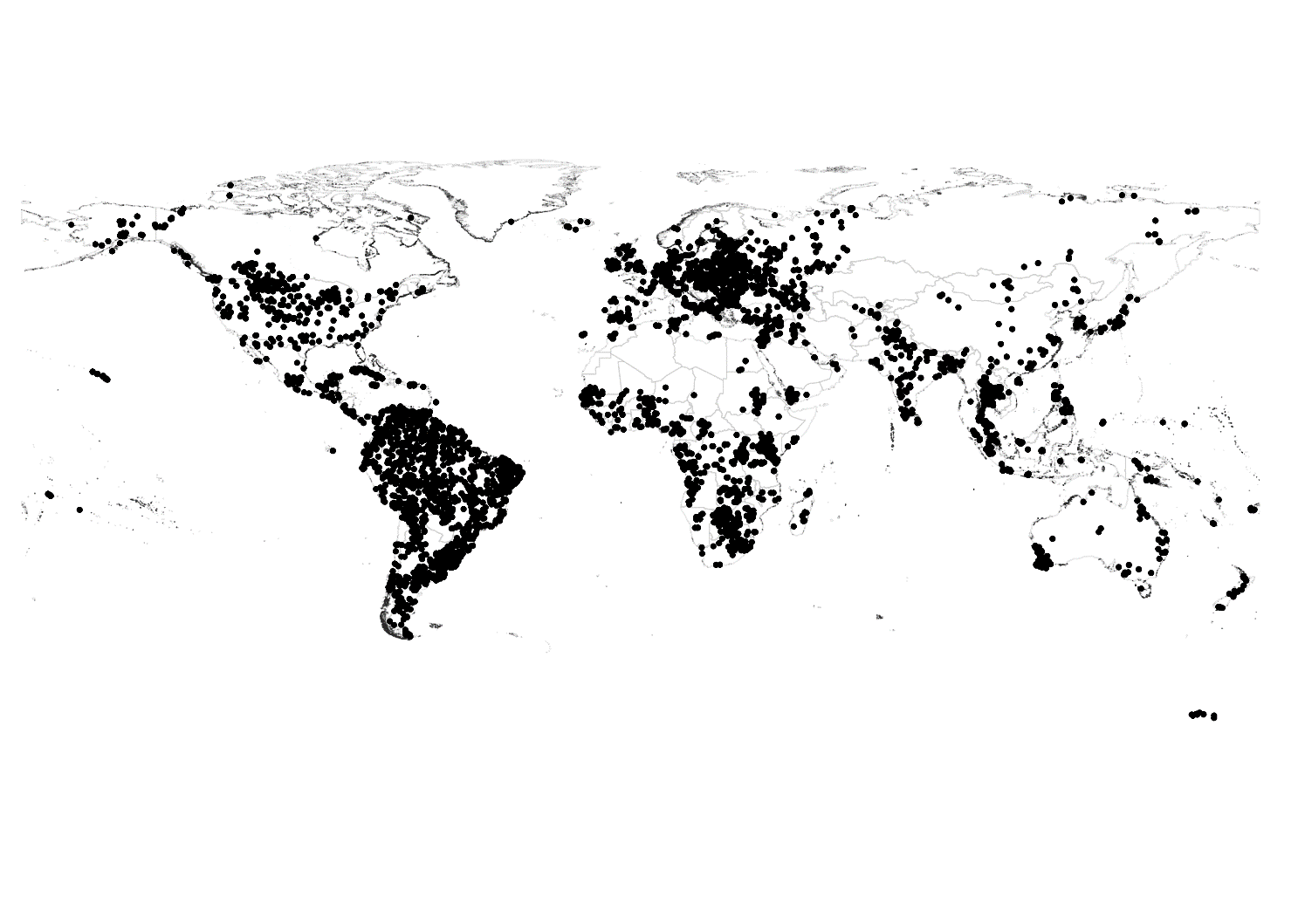


Figure S1.3: Spatial distribution of particle size distribution data in global dataset (data source: HWSD^3^)

Soil textural classes were derived from particle size distribution using United States Department of Agriculture (USDA) textural classification triangle^4^. These textural classes were Clay (denoted as Cl), Clay Loam (ClLo), Loam (Lo), Loamy Sand (LoSa), Sand (Sa), Sandy Clay (SaCl), Sand Clay Loam (SaClLo), Sandy Loam (SaLo), Silt (Si), Silty Clay (SiCl), Silty Clay Loam (SiClLo), and Silty Loam (SiLo). EC variation with soil textural classes and geographic regions are given in Figure S1.4 and Figure S1.6.


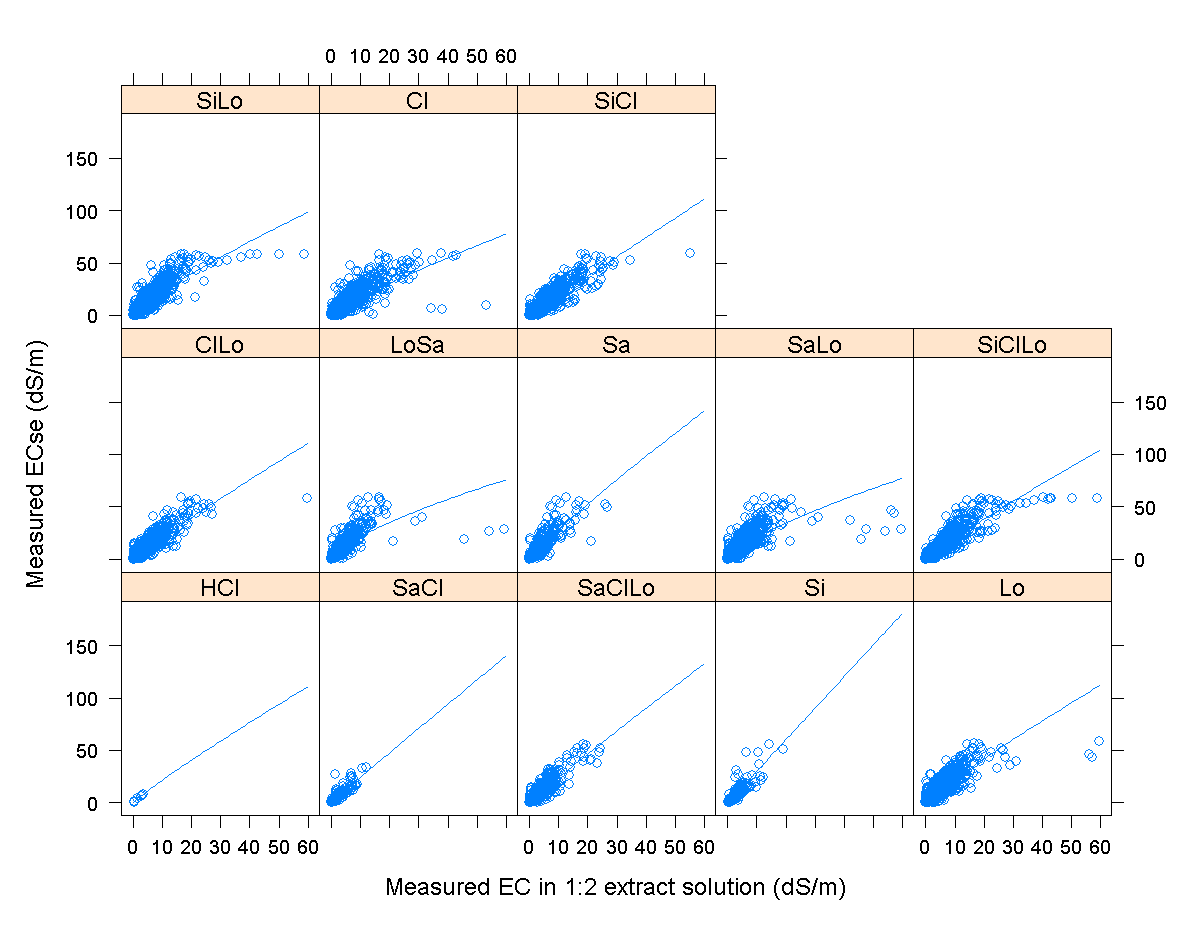


Figure S1.4: Boxplot of soil EC in different USDA textural classes

Geographic regions are Pacific, Asia, Sub-Saharan Africa (Africa), Europe (including Europe and EURASIA), Latin America and the Caribbean (LAC), Near East and North Africa (NENA), and North America (America) (Figure S1.5).


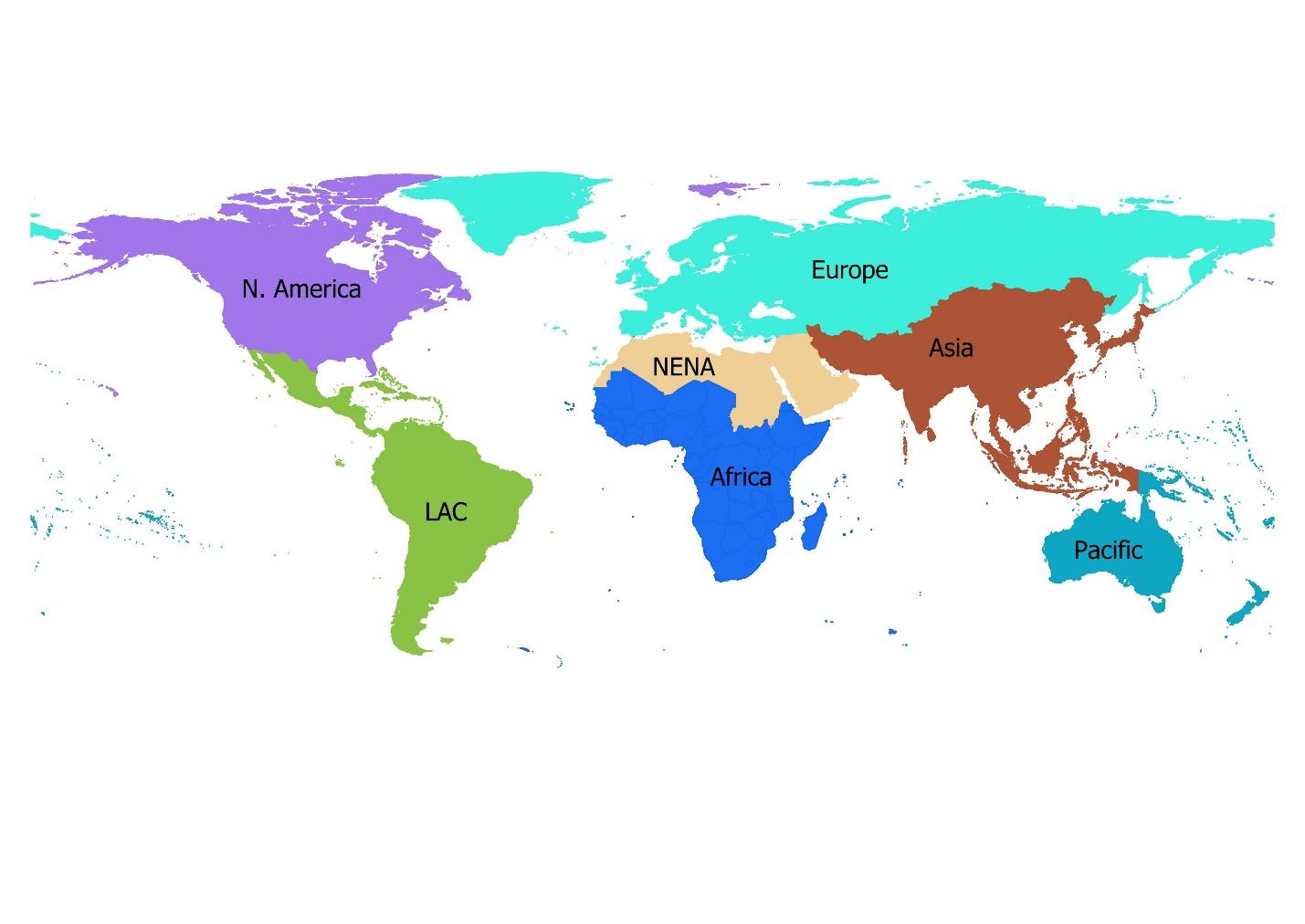


Figure S1.5: Regions of the world


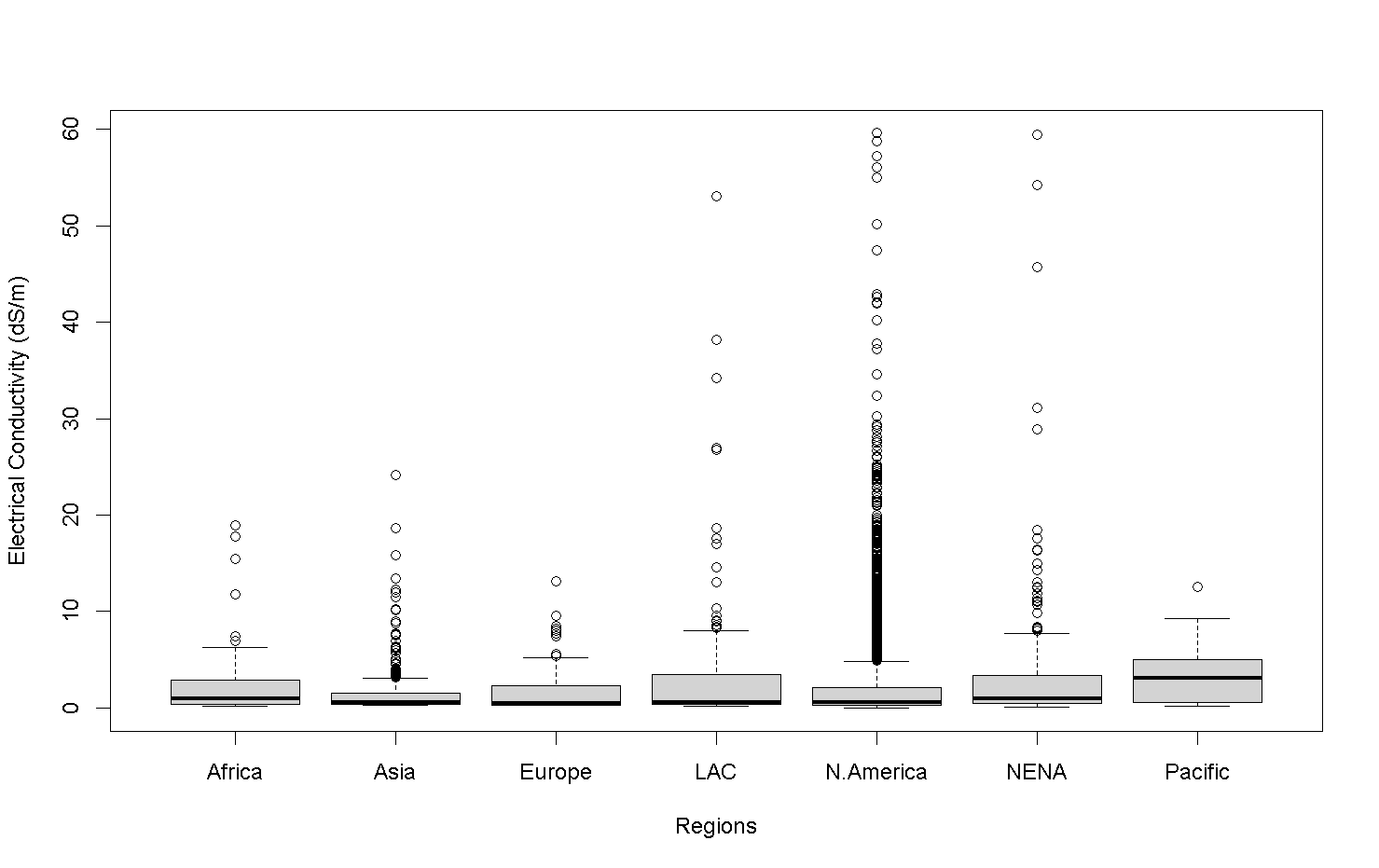


Figure S1.6: Boxplot of soil EC_1:2_ in different geographic regions of the world.

#### 2. Fitting mixed-effects harmonization models

Equation (S2.1) gives the general expression for mixed-effects harmonization model with *θ* as fixed-effects curve-fitting parameters, *b_j_* as random-effects for each of the *m* grouping variables, and *ε_i_* are random residuals.

$y_{ij}=f(x_{ij},\varphi_{i})+{g(\varepsilon}_{ij})$ *for* 1 ≤ *i* *≤ n*

$\varphi_{j}=\theta+b_{j}$ for 1 ≤ *j* ≤ *m*  (S2.1)

*f* is a function to harmonize *x* soil variables into *y, n* is the number of samples, and *g* represents a function for the residual model. Scatterplot of the SAS soil properties showed that there are many possibilities for harmonization models (Figure S2.1). In this study, seven functions were chosen to represent *f* function in Equation (S2.1) based on the orientation of the scatterplot in Figure S2.1. The models are given in Table S2.1.


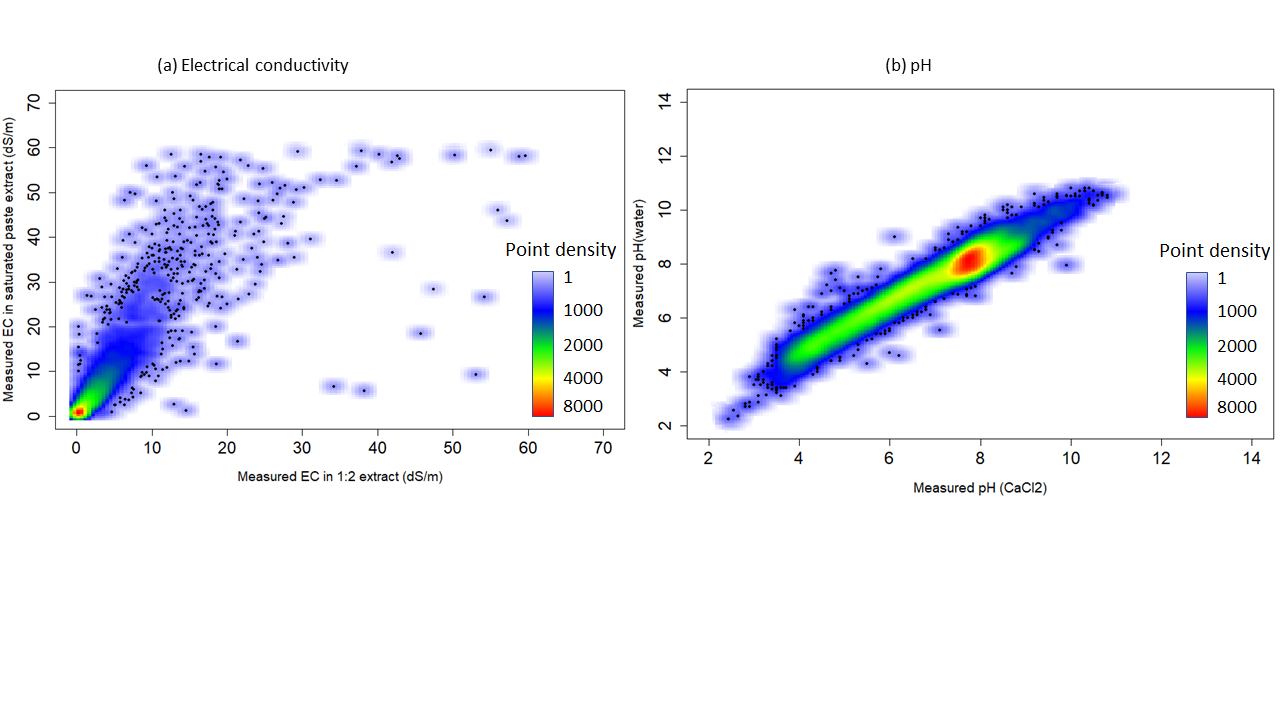


Figure S2.1: Examples of scatterplot between measured SAS soil properties

Table S2.1: Harmonization models

| Model | Model expression | Model curve-fitting parameters |
| --- | --- | --- |
| sigmoid | $y=A/\left( 1+B*exp\left( -m*x \right) \right)$ | *A, B, m* |
| power | $y=A*x^{B}$ | *A, B* |
| linear | $y=B*x+A$ | *A, B* |
| exponential | $y=A+B*\left( 1-exp\left( -m*x \right) \right)$ | *A, B, m* |
| gaussian | $y=A+B*\left( 1-exp\left( -m*x \right)^{2} \right)$ | *A, B, m* |
| polynomial | $y=A*x+B*x^{2}$ | *A, B* |
| spherical | $y=A+B*\left( m*x-\left( m*x \right)^{3} \right)$ | *A, B, m* |

Assuming the error terms are independent and identically distributed with 0 mean and *a* standard deviation and that the parameters have a **ψ** variance-covariance matrix, then for a normal distribution the harmonization model parameters can be linearly decomposed as shown in Equation (S2.2)^5^.

$\varphi_{j}=\theta+b_{j}$

$b_{j}\sim N(0,\psi)$ and $\varepsilon_{i}\sim N(0,a^{2})$ (S2.2)

The parameters of the harmonization model to be estimated are *ω* = (θ, ψ, *a*^2^) using either maximum likelihood or Bayesian approaches. This study focused on maximum likelihood approach in which the estimation of *ω* involves maximizing observed likelihood function in Equation (S2.3).

$L\left( \omega,y \right)=\int probability\left( y;\varphi;\omega\right)d\varphi$

$=\prod_{i=1}^{n} \int probability\left( \varphi_{i};\omega\right)probability\left( \varphi_{i};\omega\right)d\varphi_{i}$ (S2.3)

Equation (S2.3) may not have closed-form solution for non-linear expressions. Therefore, approximations are made either by linearizing the expression or by using a stochastic approach^6^. There are several algorithms in the literature for solving any of these approaches^7,8^. This paper used stochastic approximation expectation maximization (SAEM) algorithm that has been shown to be more efficient and accurate^9^. In this algorithm, the likelihood in Equation (S2.3) is maximized through expectation-maximization (EM) steps albeit with stochastic simulations for the expectation (E) step^10^.

#### 3. Data splitting for calibrating and validating harmonization models

Frequency distribution of EC data showed that the data were skewed toward the low values (Figure S3.1). The data were stratified according to soil textural classes and randomly split into two: calibration and validation datasets. Each stratum was recursively partitioned to establish a suitable proportion for the calibration and validation datasets. The split was started with a ratio of 1:9 (calibration: validation) and consecutively increased the calibration proportion (and decreased the validation proportion) each time during the partitioning. A combination of low residual standard error (RSE), high correlation (r^2^), and high Nash-Sutcliffe coefficient of efficiency (NSE)^11^ were used to select the appropriate data splitting strategy. 1:1 data splitting ratio seemed appropriate for EC and pH data (Figure S3.2). Frequency distribution of the split data were compared to ascertain that the calibration and validation data had similar distribution (Figure S3.3).


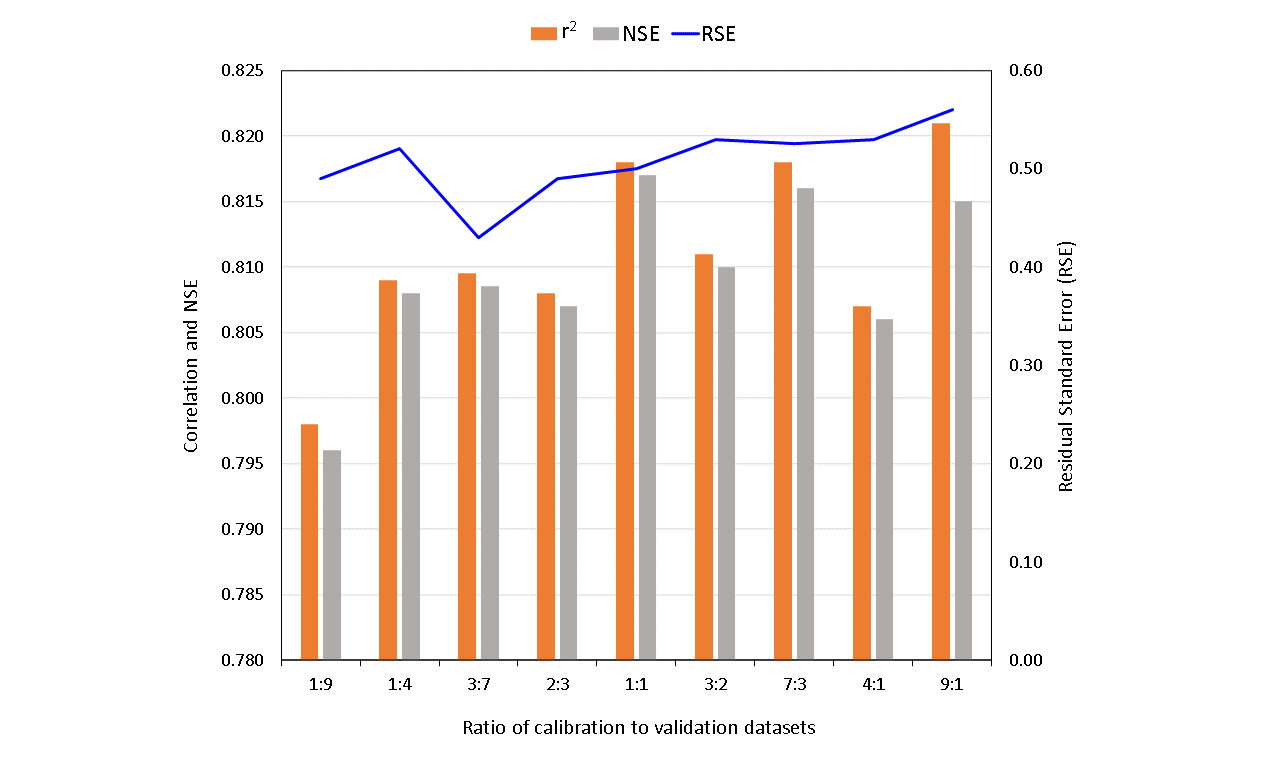


Figure S3.1: Frequency distribution of EC data


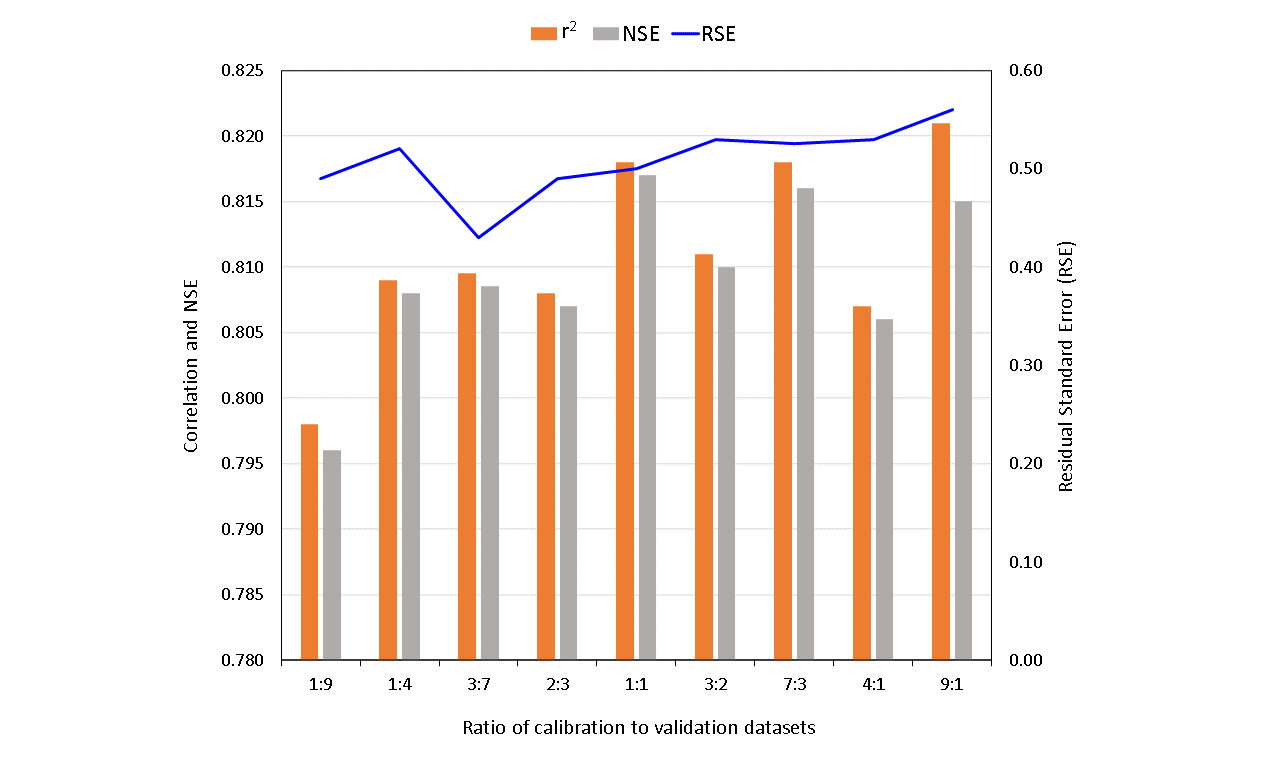


Figure S3.2: Variation of data splitting ratios and validation statistics (correlation, NSE, and RSE)


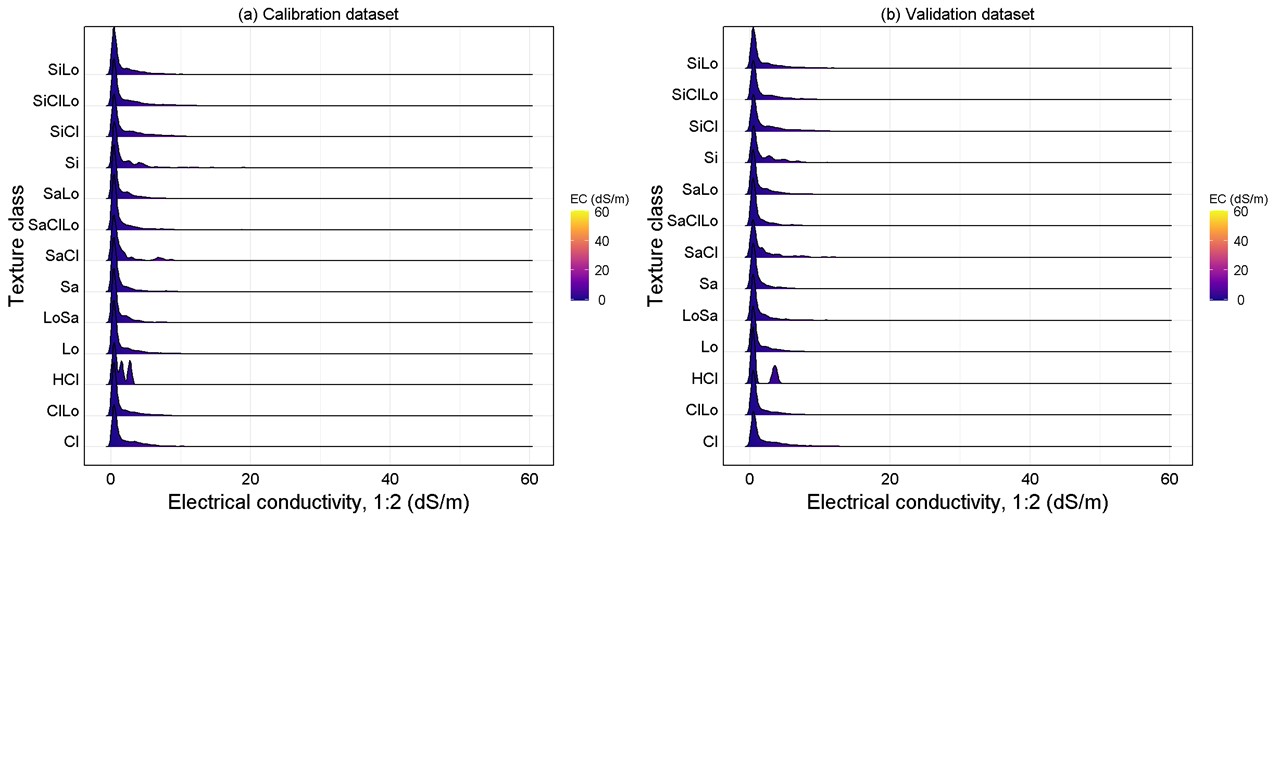


Figure S3.3: Comparison of frequency distribution of split data

#### 4. Performance evaluation of harmonization models

Table S4.1: Tested EC and pH harmonization models from the literature

| **Models for harmonizing electrical conductivity** | | | | | |
| --- | --- | --- | --- | --- | --- |
|  | Model name* | Model expression | Expression form | Model parameters | |
| 1 | Park^12^ | $y=Ax+B$ | Simple linear model | A=8.7, B=0 | |
| 2 | Ozcan^13^ | $y=Ax+B$ |  | A=5.97, B=-1.17 | |
| 3 | Chi&Wang^14^ | $y=Ax+B$ |  | A=11.68, B=-5.77 | |
| 4 | Viscounti^15^ | $y=Ax+B$ |  | A=6.53, B=-0.108 | |
| 5 | Klaustermeier^16^ | $y={10}^{A*log(x)+B}$ | Logarithmic model | A=1.256, B=0.766 | |
| 6 | USDA^17^ | $y=Ax+B$ | Simple linear model | A=4.5, B=0 | |
| 7 | He^18^ | $y=exp(Ax+B)$ | Exponential model | A=0.7, B=1.78 | |
| 8 | Kargas^19^ | $y=Ax+B$ | Simple linear model | A=1.83, B=0.117 | |
| 9 | Khorsandi^20^ | $y=Ax+B$ |  | A=5.4, B=-0.61 | |
| 10 | Sonmez^21^ | $y=Ax+B$ |  | A=8.22, B=-0.33 | |
| 11 | FAO^22^ | $y=f(x,texture,OM)$ | Linear model |  | |
| 12 | Hogg^23^ | $y=Ax+B$ | Simple linear model | A=1.75, B=-0.37 | |
| 13 | Aboukila^24^ | $y=Ax+B$ |  | A=11.74, B=-6.15 | |
| 14 | Landon^25^ | $y=Ax+B$ |  | A=6.4, B=0 | |
| 15 | Zhang^26^ | $y=Ax+B$ |  | A=1.79, B=1.46 | |
| 16 | Halder | $y=Ax+B$ |  | A=4.83, B=0.437 | |
| 17 | Shahid | $y=Ax+B$ |  | A=4.77, B=0 | |
| **Models for harmonizing pH** | | | | | |
| # | Model name* | Model expression | Model form |  | |
| 1 | Miller^27^ | $y=Ax+B$ | Simple linear model | A=0.926, B=0.901 | |
| 2 | Davies^28^ | $y=Ax+B$ |  | A=0.597, B=0.838 | |
| 3 | Brennan^29^ | $y=Ax+B$ |  | A=1.089, B=0.387 | |
| 4 | Kabala^30^ | $y=A*log(x)+B$ |  | A=11.58, B=-1.95 | |
| 5 | Sadovski^31^ | $y=Ax+B$ |  | A=0.888, B=1.535 | |
| 6 | Ahern^32^ | $y=Ax+B$ |  | | A=1.083, B=0.404 |

Table S4.2: More information about tested EC and pH harmonization models

| # | Model name* | Area where it was developed | Soil solution to harmonize | |
| --- | --- | --- | --- | --- |
| **Models for harmonizing electrical conductivity** | | | | |
|  |  |  | Texture groups | soil:water ratio |
| 1 | Park^12^ | Korea | No | 1:5 |
| 2 | Ozcan^13^ | Turkey | No, Yes | 1:5, 1:2.5 |
| 3 | Chi&Wang^14^ | China | No | 1:5 |
| 4 | Viscounti^15^ | Spain | No | 1:5 |
| 5 | Klaustermeier^16^ | USA | No | 1:5 |
| 6 | USDA^17^ | USA | No | 1:5, 1:2 |
| 7 | He^18^ | USA | No | 1:5 |
| 8 | Kargas^19^ | USA | No | 1:5 |
| 9 | Khorsandi^20^ | Iran | No | 1:5 |
| 10 | Sonmez^21^ | Turkey | Yes | 1:5, 1:2.5 |
| 11 | FAO^22^ | Global | Yes | 1:5, 1:2, 1:2.5 |
| 12 | Hogg^23^ | Canada | Yes | 1:2 |
| 13 | Aboukila^24^ | Egypt | Yes | 1:5 |
| 14 | Landon^25^ | Pacific | No | 1:1, 1:3, 1:5 |
| 15 | Zhang^26^ | USA | No | 1:1 |
| 16 | 7 Mixed effects (ME) models (Table S2.1) | Global | Yes | 1:5, 1:2, 1:2.5 |
| **Models for harmonizing pH** | | | | |
| # | Model name* | Area where it was developed | pH (CaCl_2_) | pH (KCl) |
| 1 | Miller^27^ | USA | Yes |  |
| 2 | Davies^28^ | England | Yes |  |
| 3 | Brennan^29^ | Australia | Yes |  |
| 4 | Kabala^30^ | Spain |  | Yes |
| 5 | Sadovski^31^ | Bulgaria |  | Yes |
| 6 | Ahern^32^ | Australia | Yes |  |
| 7 | Minasny^33^ | Australia | Yes |  |
| 8 | 7 Mixed effects (ME) models (Table S2.1) | Global | Yes | Yes |

* Model name is for use in this article to identify the model

To facilitate further robust evaluation of the harmonization models, the validation dataset was grouped according to geographic regions and into high (EC≥8 dS/m and pH≥7) and low values (EC<8 dS/m and pH<7). Performance of the models in the high and low data ranges is shown in figure S4.1.


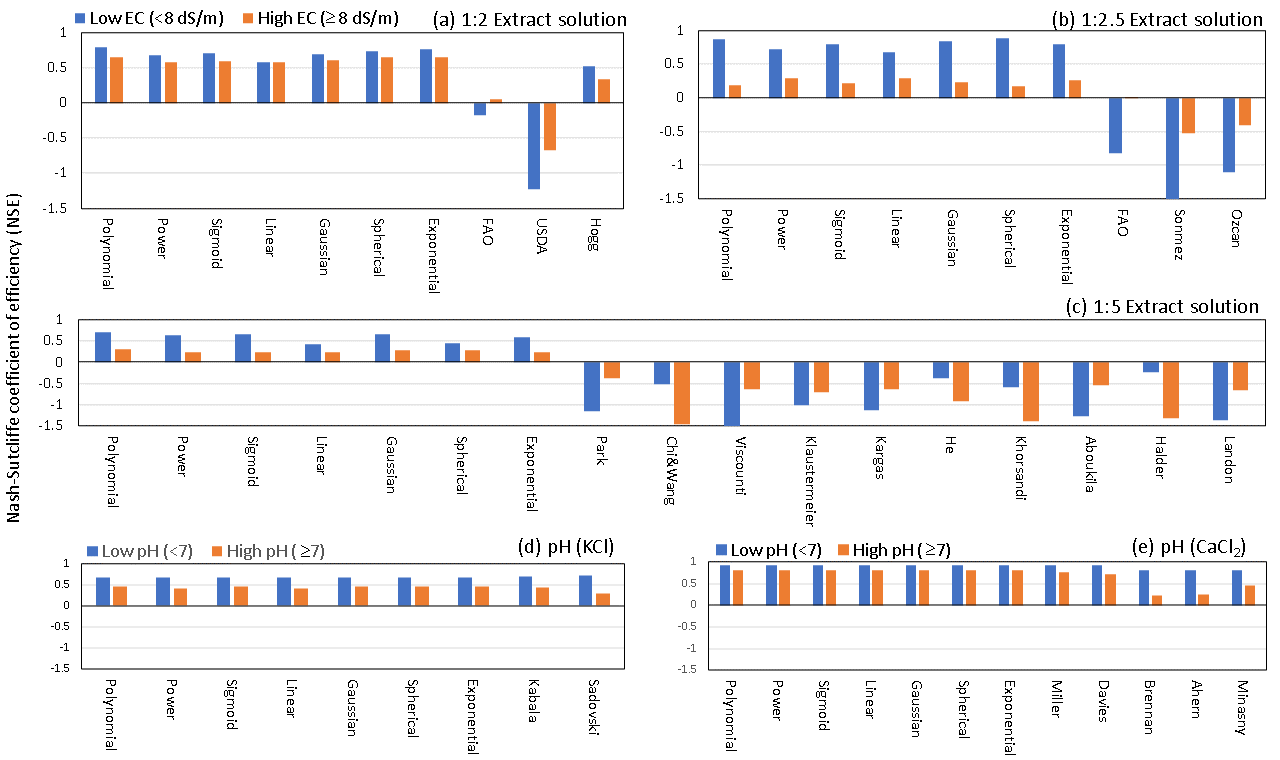


Figure S4.1: NSE comparison of harmonization models for low/high PH or EC values


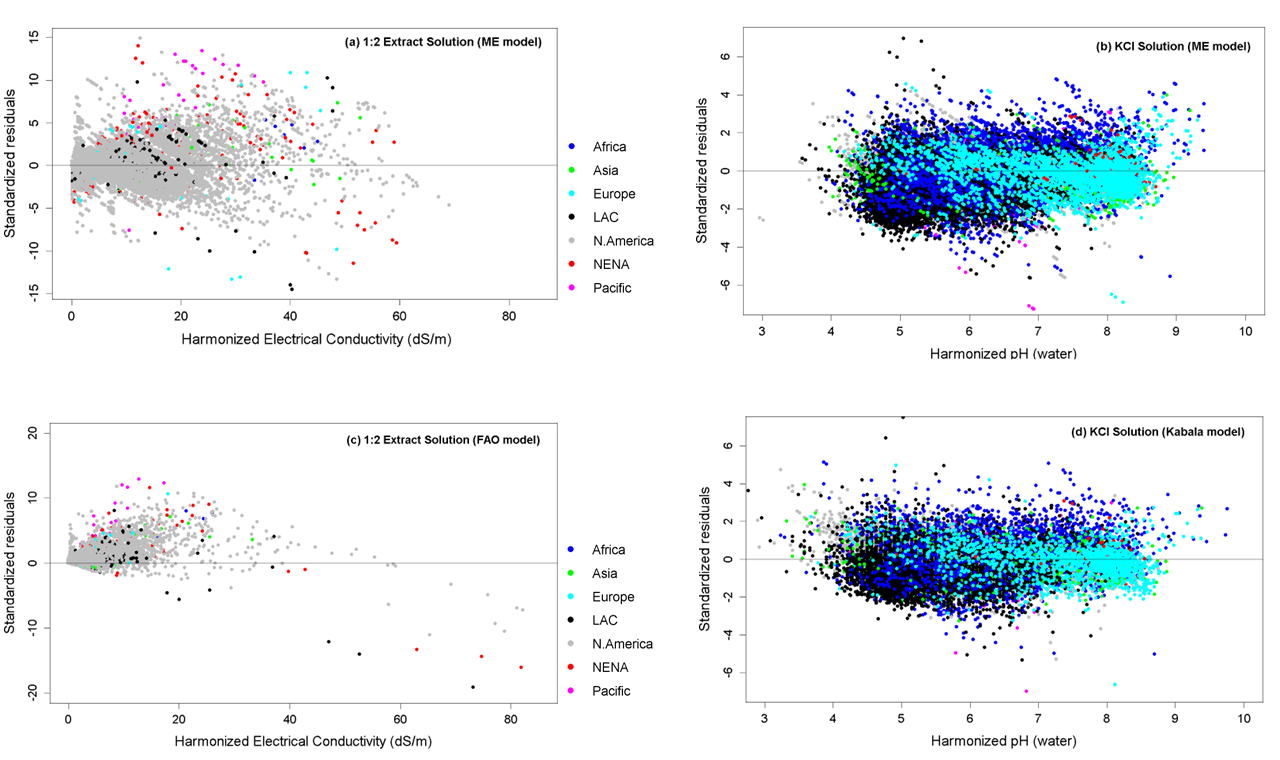


Figure S4.2: Examples of plot of standardized residuals for harmonization models

Graphical illustrations of standardized residuals showed that most models from the literature had high variations in EC harmonization for values greater than 8 dS/m (Figure S4.2). Plots of standardized residuals for these models had increasing residual variance with increasing EC values. This implies that the models did not adequately represent high EC values, which could lead to high uncertainties in mapping high SAS intensity classes. Standardized residual plots for the ME models showed less heteroscedasticity than models from the literature. They were able to model some of the residual variance through random-effects modelling, which minimized the proportion of unexplained variance and consequently improving the harmonization. Consequently, the plots were used to identify unique observations such as outliers (Figure S4.3). In the EC data with 1:2 extract solution, the ME model identified some samples from Jordan, Oman, Antarctica, Puerto Rico, Chile, United Arab Emirates (UAE), United States of America (USA), Russia, Mongolia, and Argentina as possible outliers. In the EC data with 1:2.5 extract solution, possible outliers were identified in Mali while in the 1:5 extract solution possible outliers come from northwest Australia and northeast Nigeria.


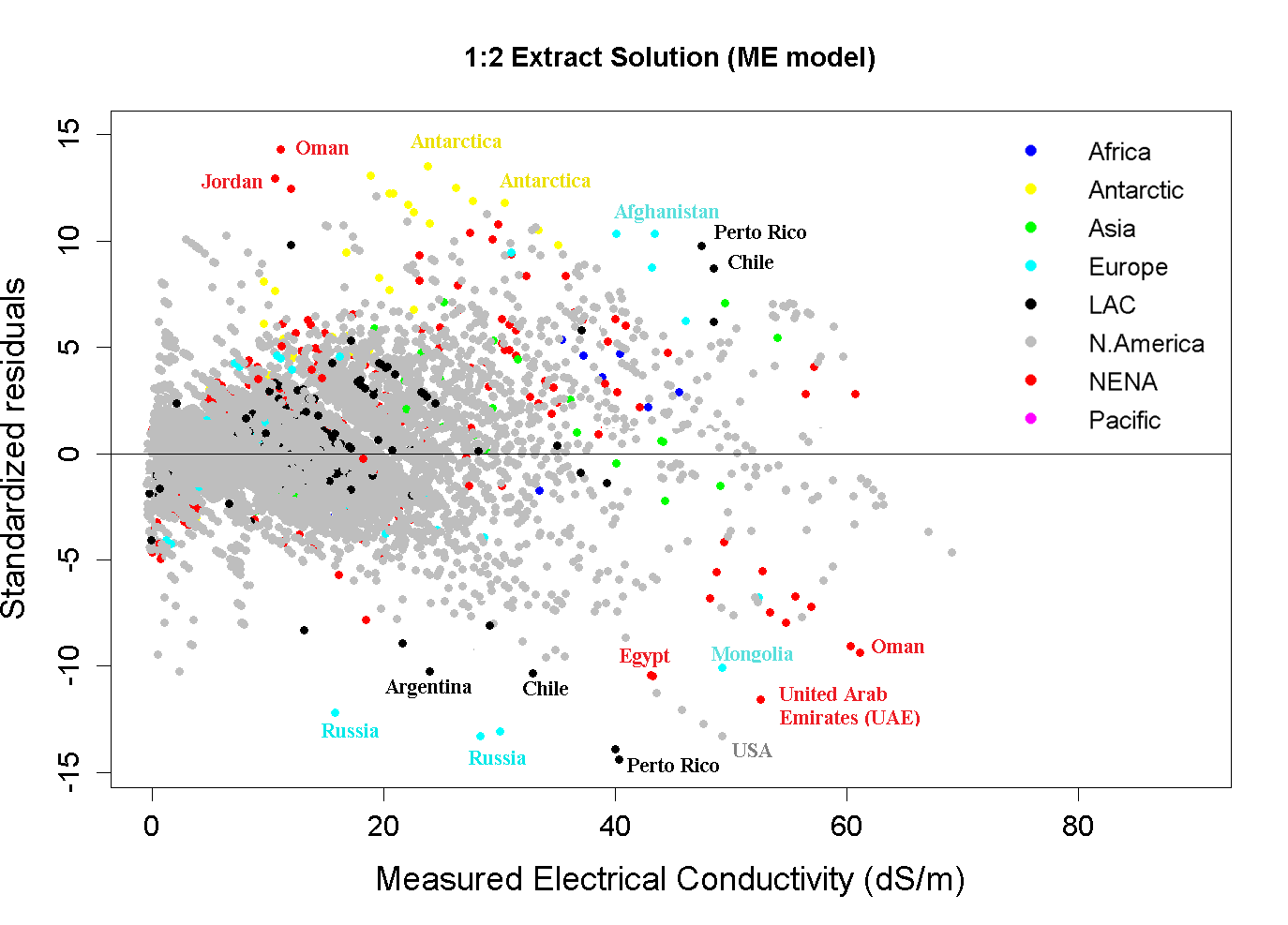


Figure S4.3: Identification of outliers from a standardized residual plot from ME model


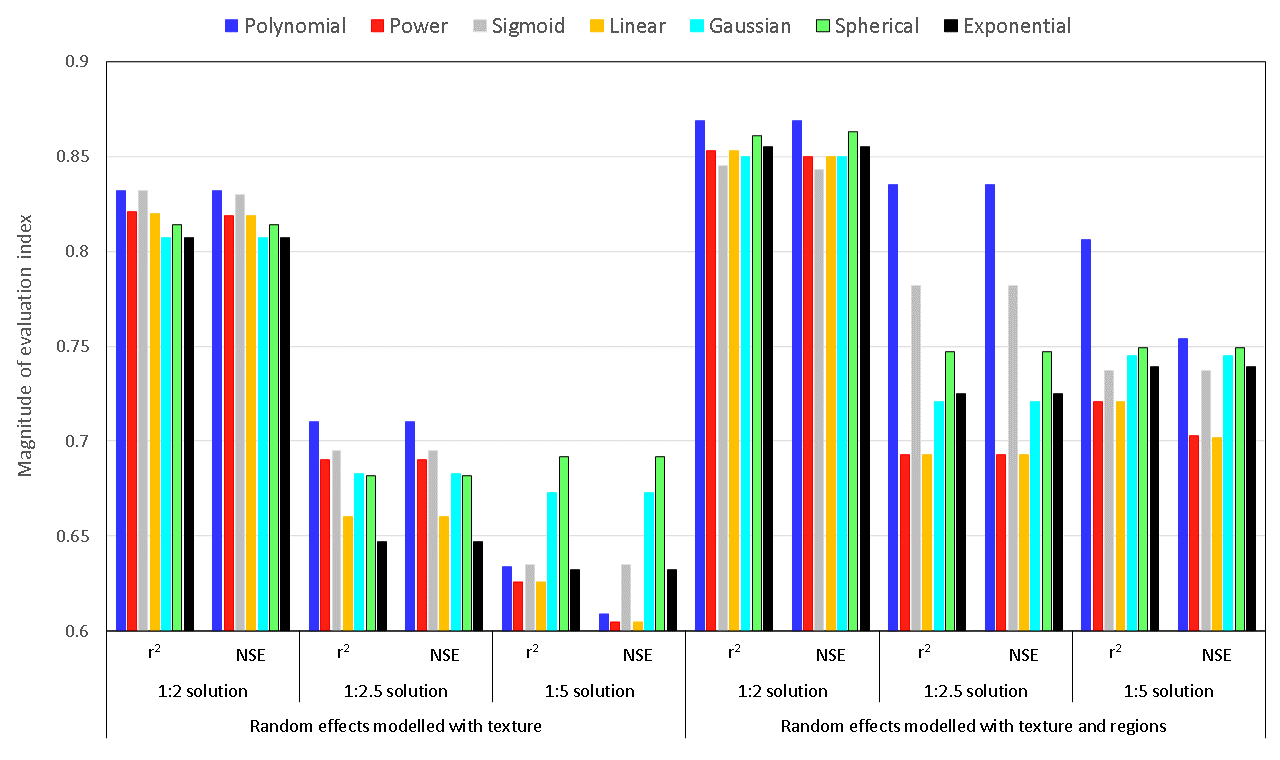


Figure S4.4: Comparison of NSE and r^2^ for ME models with different random-effects modelling


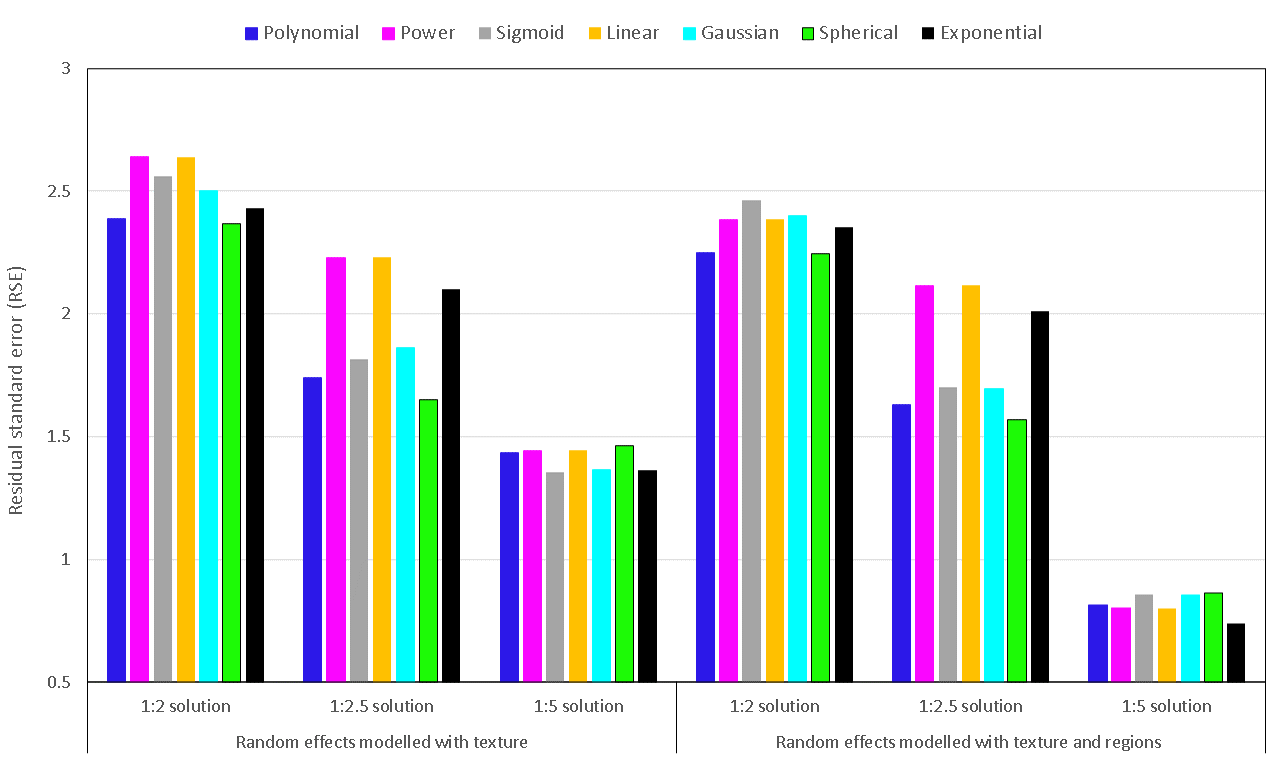


Figure S4.5: Comparison of RSE for ME models with different random-effects modelling

#### 5. Harmonization service

The service is a set of computer codes for harmonizing EC and pH and codes for providing relevant information on available harmonization models and global data density (Table S5.1). These codes are contained in the *soilassessment* package of R^34^ and are periodically updated.

Table S5.1: Categories of services and available models in SAS harmonization

| Main service category | Service sub-category | Function name | Function role |
| --- | --- | --- | --- |
| SAS data Harmonization service | Mixed-effects harmonization functions | ME_ECharm | Harmonization of EC using mixed effects |
|  |  | ME_PHharm | Harmonization of pH using mixed effects |
|  | Harmonization functions for expressions in the literature | ECconversion1 | Harmonization of EC by soil texture groups |
|  |  | ECconversion2 | Harmonization of EC without texture groups |
|  |  | PHconversion | Harmonization of pH without texture groups |
|  | Functions to facilitate own harmonization model development | conversion | Developing model between EC_se_ and other EC |
|  |  |  | Developing model between EC_se_ and bulk EC |
|  |  |  | Developing model between pH and pH (sensor) |
|  |  | pedoTransfer | Developing pedotransfer models between EC_se_ and other soil properties (e.g. infrared reflectance, exchangeable cations, total soluble salts, electromagnetic induction, calcium carbonate, etc.) |
| SAS information service | SAS model information | SASmodels | Harmonization models |
|  | Regional performance of harmonization models | ECharm_Info | Harmonization performance status for EC models |
|  |  | PHharm_Info | Harmonization performance status for pH models |
|  | Data density status for available global SAS data | SASdata_densityInfo | Information on global density of available SAS data |
|  |  | DataAvailabilityIndex | SAS data availability index showing number of samples per square grid cell |

**5.1 Examples of how to use the service in SAS data harmonization**

**5.1.1 Example data for demonstration**

*library(soilassessment)*

*ECdata=data.frame(EC=c(1,0.34,5.07,12.17, 2.219),*

*OC=c(0.25,0.8,0.07,0.01,0.45),*

*Clay=c(12.2,33.1,8.9,21.4,17.6),*

*TEX=c("Cl","SaCl","LoSa", "SiCl","SaClLo"))*

*PHdata=data.frame(ph=c(1.6,8.3,5.7,12.1, 2.2),tex=c("Cl","SaCl","LoSa", "Si","SaClLo"))*

**5.1.2 Mixed-effects harmonization**

Harmonization of EC using the service requires: 1) EC data from 1:2 or 1:2.5 or 1:5 extract solution, 2) soil textural class (according to USDA classification), 3) conversion model (Table S2.1), and 4) soil-water mix ratio for extract solution. Harmonization of pH requires: 1) pH data from KCl or CaCl_2_ solution, 2) soil textural class (according to USDA classification), 3) conversion model (Table S2.1), and 4) pH solution (KCl or CaCl_2_). The following are example scripts for EC and pH harmonization:

*ECdata$ME=ME_ECharm(ECdata$EC,ECdata$TEX,"power","1:5")*

*PHdata$ME=ME_PHharm(PHdata$ph,PHdata$tex,"exponential","kcl")*

**5.1.3 Harmonization using models in the literature**

The models are further grouped into models that use textural classes and those that do not use textural classes. EC harmonization requires: 1) EC data from a variety of extract solutions depending on the model, 2) optional soil textural class (according to USDA classification) in case the model requires textural class, 3) conversion model (Table S2.1), and 4) soil-water mix ratio for extract solution. pH harmonization also differs depending on the solution used and requires

*ECdata$FAO=ECconversion1(ECdata$EC, ECdata$TEX, "FAO","1:5", ECdata$OC,ECdata$Clay)*

*ECdata$USDA=ECconversion2(ECdata$EC,"USDA","1:5")*

*PHdata$kabala=PHConversion(PHdata$ph,"kabala","kcl")*

*PHdata$ahern=PHConversion(PHdata$ph,"ahern","cacl2")*

**5.1.4 Building own harmonization model**

The harmonization is based on a subset of data with concurrent measurements of ECse or pH(water) and non-standard EC or pH values or other soil properties for model development and using the model to harmonize measurements without the standard values.

*#part of data with concurrent measurements for development of harmonization model*

*x=as.vector(c(0.800,2.580,0.980,0.532,1.870, 18.500,0.430,0.302,0.345,2.700,3,4.1))*

*y=as.vector(c(17.88, 6.43, 3.83, 7.18, 6.64, 14.83, 4.19, 7.31, 3.21, 18.41,2.9,5.3))*

*xy=as.data.frame(cbind(x,y))*

*names(xy)=c("ECa", "EC")*

*plot(EC~ECa,xy)*

*EC3.ml=nls(EC~conversion(ECa,A,B, "log"), start=c(A=0.1, B=0.8), data=xy)*

*# Part of data to harmonize*

*ECdata2=data.frame(ECa=c(12.33, 6.01, 11.99, 0.18, 5.21, 7.18, 9.02, 8.98, 5.61, 23.5,13, 14.02, 10.82, 101.15,2.9,6.6))*

*ECdata2$EC=predict(EC3.ml,ECdata2)*

*#pedo-transfer with other soil properties*

*xy$Clay=runif(12, 1,100)/3*

*xy$Silt=runif(12, 1,100)/2*

*xy$Sand=100-(xy$Clay+xy$Silt)*

*xy$pH=c(7.97,2.92,4.11,4.29,4.43,11.3,1.39,6.39,1.13,11.16,3.07,6.44)*

*xy$OC=c(0.970,0.47,0.256,0.674,0.354,0.914,0.402,0.331,0.277,1.14,0.223,0.631)*

*xy$EC1=xy$EC; xy$EC1[7:nrow(xy)]=NA*

*EC1.lm=pedoTransfer("randomforest",subset(xy,!is.na(xy$EC1)),EC1, Clay,Sand,Silt,OC,pH)*

*xy$ECse=ifelse(is.na(xy$EC1),predict(EC1.lm,xy),xy$EC1)*

**5.2. SAS information service**

5.2.1 SAS Information

SAS information is provided for available SAS harmonization models and performance index for different harmonization models in the main regions of the world. Input variable for SAS model information is the data category and extract solution for performance indices. The following scripts illustrate how to obtain the SAS information in the service.

*SASmodels(“ec) #Models for EC harmonization*

*PHharm_Info("kcl") #Performance indices for different PH models in various regions of the world*

5.2.2 Global SAS data availability

Data availability in the global database is given in terms of global density of EC and pH data points and data availability in the global database at a given extent/area of interest. The input variable for the function for data availability is the data category. The function for data availability information requires: 1) the extent for area of interest, 2) user-defined scale (or spatial resolution) for estimating density of points, 3) coordinate reference system, and 4) the type of SAS data category (e.g., ec or ph). The following scripts demonstrate how the information can be obtained from the service

*SASdata_densityInfo("ec")*

*SASglobeData("ec2.5","KEN")*

*x <- c(20.02,25.69,25.69,20.02)*

*y <- c(-28.40,-32.76,-32.76,-34.84)*

*yx=data.frame(cbind(x, y))*

*CRs=CRS("+init=epsg:4326")*

*Data=SASglobeData("ph","ZAF")*

*coordinates(Data)=~Longitude+Latitude*

*proj4string(Data)=CRs*

*Index=DataAvailabilityIndex(yx,60,CRs,Data)*

#### 6. Application of harmonization service in national SAS information development

**6.1 Input soil data and covariates for producing spatial maps of SAS information**

Input soil data from the case study countries were EC, pH, exchangeable sodium ions (Na^+^), exchangeable cation capacity (CEC), and particle size distribution (Table S6.1 and S6.2). These soil properties were measured in soil laboratories on samples collected from the field at varied depth intervals between 0 and 250 cm.

Table S6.1: Summary of soil data from 30 case-study countries

| Region | Country | Country-level data | | | Data available in global dataset | | | pH  solution |
| --- | --- | --- | --- | --- | --- | --- | --- | --- |
|  |  | Sampling points | Sampling dates | Soil extract^*^ | Sampling points | Sampling dates | Soil extract |  |
| Caribbean | Cuba | 3,317 | 1970-1973 | 1:2.5 | 47 | 1970-1978 | 1:2.5 | Water |
|  | Jamaica | 90 |  | 1:5 | 30 | 1979-1983 | 1:5 | Water |
|  | Trinidad and Tobago | 122 | 1960-1970 | SP | 2 | 1960 | 1.5 | Water |
| Asia | Bangladesh | 74 | 2000-2009 | 1:5 | 17 | 1971 | 1:5 | Water |
|  | Thailand | 117 | 2000-2010 | SP | 50 | 1978-1988 | SP | KCl |
|  | Cambodia | 316 | 2019-2020 | 1:5 | - | - | - | Water |
|  | Pakistan | 37,957 | 2014-2018 | 1:5 | 38 | 1984-1989 | 1:5 | Water |
|  | Afghanistan | 191 | 2018-2019 | 1:2.5 | 3 | 1962 | 1:2.5 | Water |
|  | India | 3,491 | 2012-2019 | 1:2, SP | 138 | 1989-1990 | 1:2 | Water |
|  | Myanmar | 217 | 2010-2020 | SP | - | - | - | - |
|  | Philippines | 62 | 1985-2015 | SP | 22 | 1982-1987 | 1:2 | KCl |
| Pacific | Western Samoa | 72 | 1963-2019 | 1:5 | 28 | 1985 | 1:5 | KCl |
|  | Papua New Guinea | 31 | 1972-1980 | SP | 17 | 1982-1983 | 1:5 | Water |
| Europe | Italy | 13,784 | 1969-2019 | SP | 1,641 | 2015 | 1:5 | KCl |
|  | Germany | 4,954 | 2006-2018 | 1:5 | 4,338 | 2015 | 1:5 | Water |
| Africa | South Africa | 2,000 | 2007-2009 | SP | 874 | 1962-1983 | SP | KCl |
|  | Nigeria | 428 | 1990-2019 | 1:5 | 402 | 1962-2003 | 1:5 | KCl |
|  | Kenya | 350 | 1990-2003 | 1:2.5 | 158 | 1989-2003 | 1:2.5 | Water |
|  | Rwanda | 1,741 | 201-2017 | 1:2 | 88 | 1963-1993 | 1:2 | Water |
|  | Senegal | 2,664 | 1910-2017 | SP | 149 | 1956-2003 | SP | KCl |
| Near East | Oman | 590 | 1990-1999 | 1:5 | 84 | 1982-1990 | SP | KCl |
|  | Jordan | 3,740 | 1990-2010 | 1:5 | 43 | 1982-1992 | 1:5 | Water |
| Central Asia | Kazakhstan | 5,530 | 2010-2014 | SP | 12 | 1972-1984 | 1:5 | Water |
| Latin America | Peru | 2,266 | 1990-2020 | SP | 150 | 1974-1991 | 1:2.5 | Water |
|  | Bolivia | 12,608 | 1964-2016 | 1:5 | 86 | 1974-1995 | 1:2 | Water |
|  | Ecuador | 462 | 2009-2015 | SP | 76 | 1970-1988 | 1:2.5 | Water |

^*^SP – saturated paste extract

The measurement methods used are summarized in Table S6.1 and are like those given in the documentation of global databases^1,3^. Cation Exchange Capacity (CEC) and exchangeable sodium ions (Na^+^) were used to calculate Exchangeable Sodium Percent (ESP) as shown in Equation S6.1

$ESP= \frac{{Na}^{+}}{CEC}*100$ (S6.1)

Table S6.2: Methods for measurement of soil properties in the case studies

| Region | Country | pH | EC | Sand | Silt | Clay | Na^+^ | CEC |
| --- | --- | --- | --- | --- | --- | --- | --- | --- |
|  |  | - | dS/m | % | % | % | (cmol/mg) | (cmol/mg) |
| Caribbean | Cuba | * | + | ο | ο | ο | ⊕ | ⊕ |
|  | Jamaica | * | + | ο | ο | ο | ⊕ | ⊕ |
|  | Trinidad | * | + | ο | ο | ο | ⊕ | ⊕ |
| Asia | Bangladesh | * | + | ο | ο | ο | ⊕ | ⊕ |
|  | Thailand | * | + | ο | ο | ο | ⊕ | ⊕ |
|  | Cambodia | * | + | ο | ο | ο | ⊕ | ⊕ |
|  | Pakistan | * | + | ο | ο | ο | ⊕ | ⊕ |
|  | Afghanistan | * | + | ο | ο | ο | ⊕ | ⊕ |
|  | India | * | + | ο | ο | ο | ⊕ | ⊕ |
| Pacific | Samoa | * | + | ο | ο | ο | ⊕ | ⊕ |
|  | PN Guinea | * | + | ο | ο | ο | ⊕ | ⊕ |
| Europe | Italy | * | + | ο | ο | ο | ⊕ | ⊕ |
|  | Germany | * | + | ο | ο | ο | ⊕ | ⊕ |
| Africa | South Africa | * | + | ο | ο | ο | ⊕ | ⊕ |
|  | Nigeria | * | + | ο | ο | ο | ⊕ | ⊕ |
|  | Kenya | * | + | ο | ο | ο | ⊕ | ⊕ |
|  | Madagascar | * | + | ο | ο | ο | ⊕ | ⊕ |
|  | Ethiopia | * | + | ο | ο | ο | ⊕ | ⊕ |
|  | Burkina Faso | * | + | ο | ο | ο | ⊕ | ⊕ |
|  | Rwanda | * | + | ο | ο | ο | ⊕ | ⊕ |
|  | Senegal | * | + | ο | ο | ο | ⊕ | ⊕ |
| Near East & North Africa (NENA) | Oman | * | + | ο | ο | ο | ⊕ | ⊕ |
|  | Morocco | * | + | ο | ο | ο | ⊕ | ⊕ |
|  | Jordan | * | + | ο | ο | ο | ⊕ | ⊕ |
|  | Sudan | * | + | ο | ο | ο | ⊕ | ⊕ |
| EURASIA | Kazakhstan | * | + | ο | ο | ο | ⊕ | ⊕ |
|  | Mongolia | * | + | ο | ο | ο | ⊕ | ⊕ |
| Latin America | Peru | * | + | ο | ο | ο | ⊕ | ⊕ |
|  | Bolivia | * | + | ο | ο | ο | ⊕ | ⊕ |
|  | Ecuador | * | + | ο | ο | ο | ⊕ | ⊕ |

* - pH meter, + - EC meter, ο - hydrometer method, ⊕ - Atomic absorption spectrophotometer

Table S6.3: Summary of covariates for modelling harmonized SAS input data in case-study countries

| Covariates | | Description and source |
| --- | --- | --- |
| Parent material | Geology | National geology maps |
|  | Soil types | National soil maps |
| Land use/ Land cover | | ESA Globe Cover between 2005 and 2015^†^ |
| Climate | Precipitation | Mean annual rainfall amounts^35^ |
|  | Temperature | Minimum and maximum annual air temperature^36^ |
| Relief | Elevation | Shuttle Radar Topography Mission 1 Arc-Second Global Digital Elevation Model (DEM)^37^ |
|  | Relief parameters | Relief parameters derived from DEM: curvature, slope, slope-length (ls), valley-depth (valley), elevation (dem), channel depth (cnbl)^38^ |
| Remote sensing images | | Annual mean surface reflectance from Moderate Resolution Imaging Spectroradiometer (MODIS) between 2000 and 2020^39^ |
|  |  | Remote sensing indices of salinity^35,40^ |

†<http://due.esrin.esa.int/page_globcover.php> (accessed in June 2021)

**6.2 Developing harmonized spatial maps of national SAS information**

Three-step approach was followed for developing SAS information^35^ (Figure S6.1). It was used in each case study country to develop long-term average SAS information (ECse, pH(water), ESP, and SAS intensity classes) for 0-30 cm and 30-100 cm soil depths. The input soil data (EC, pH, and ESP) for these two soil depths were obtained by use of depth-integrating splines on soil profile data^41^. Two depths were chosen to represent topsoil (0-30 cm) and subsoil (30-100 cm).


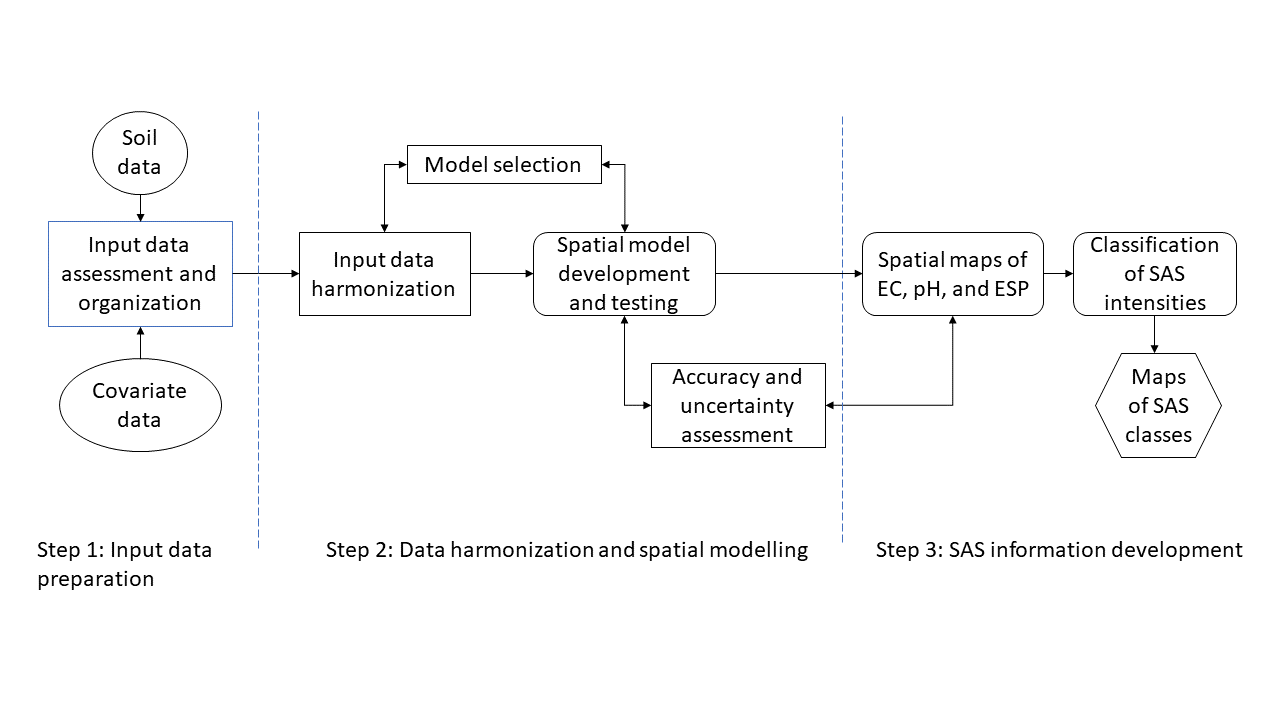


Figure S6.1: Framework for three-step approach for developing SAS information

Machine learning was used to develop models between harmonized soil properties and SAS covariates (Table S6.3) and then applying the model to produce spatial distribution (maps) of the soil properties. Commonly used machine learning algorithms for SAS mapping^35,42^ were evaluated and the most suitable algorithm for spatial distribution of soil properties selected. The selection was based on the lowest bias and root mean square error (RMSE) and highest r^2^ and Nash-Sutcliffe coefficient of efficiency (NSE). The resultant soil property maps were classified into SAS intensities according to FAO SAS classification scheme^35,43^. The accuracy of SAS classification was evaluated using tau agreement index^44^.

SAS intensity classes produced by different SAS harmonization models were compared. Most models with low prediction accuracy showed low tau agreement index, which was an indication of high SAS misclassification rate. For example, SAS classes produced by three different EC harmonization models (ME polynomial, FAO, and Ozcan models) were compared. The comparison showed that ME polynomial harmonization model had the highest overall tau index for all classes (Figure S6.2). Ozcan harmonization model produced the lowest tau index.


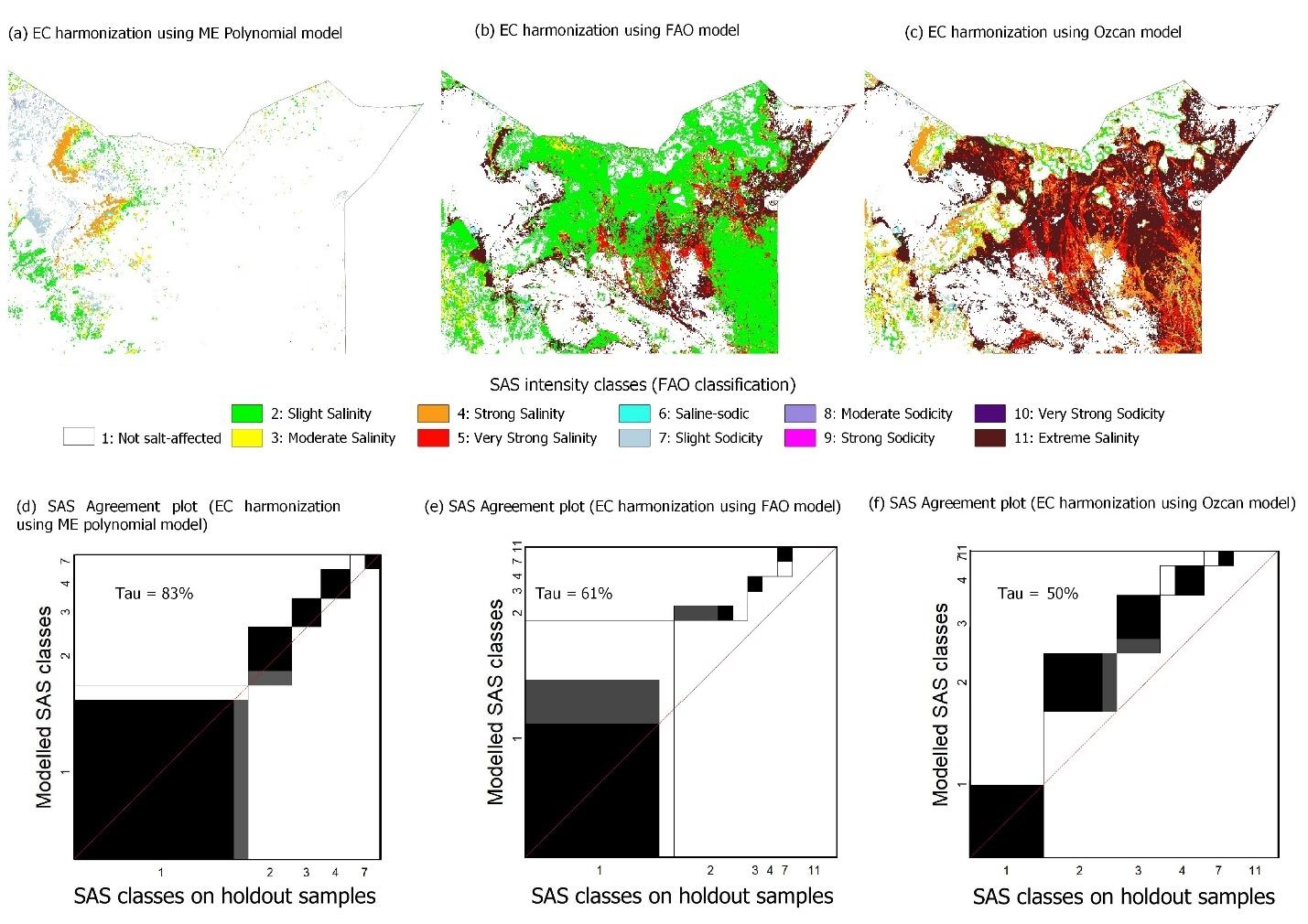


Figure S6.2: Spatial distribution of SAS maps based on different EC harmonization models

Further analysis was done for the uncertainty of spatial prediction of EC and SAS classification. Uncertainty analysis for EC was done using the bootstrap method^45,46^. In SAS classification, it was analyzed using the Monte Carlo method for uncertainty propagation^47^. The analyses showed relatively high uncertainties in areas with poor representation of measured samples (an example of EC uncertainty map in northeastern Kenya is shown in Figure S6.3a). In addition, some models with poor harmonization of EC also produced high uncertainties in SAS misclassified areas (Figure S6.3b).


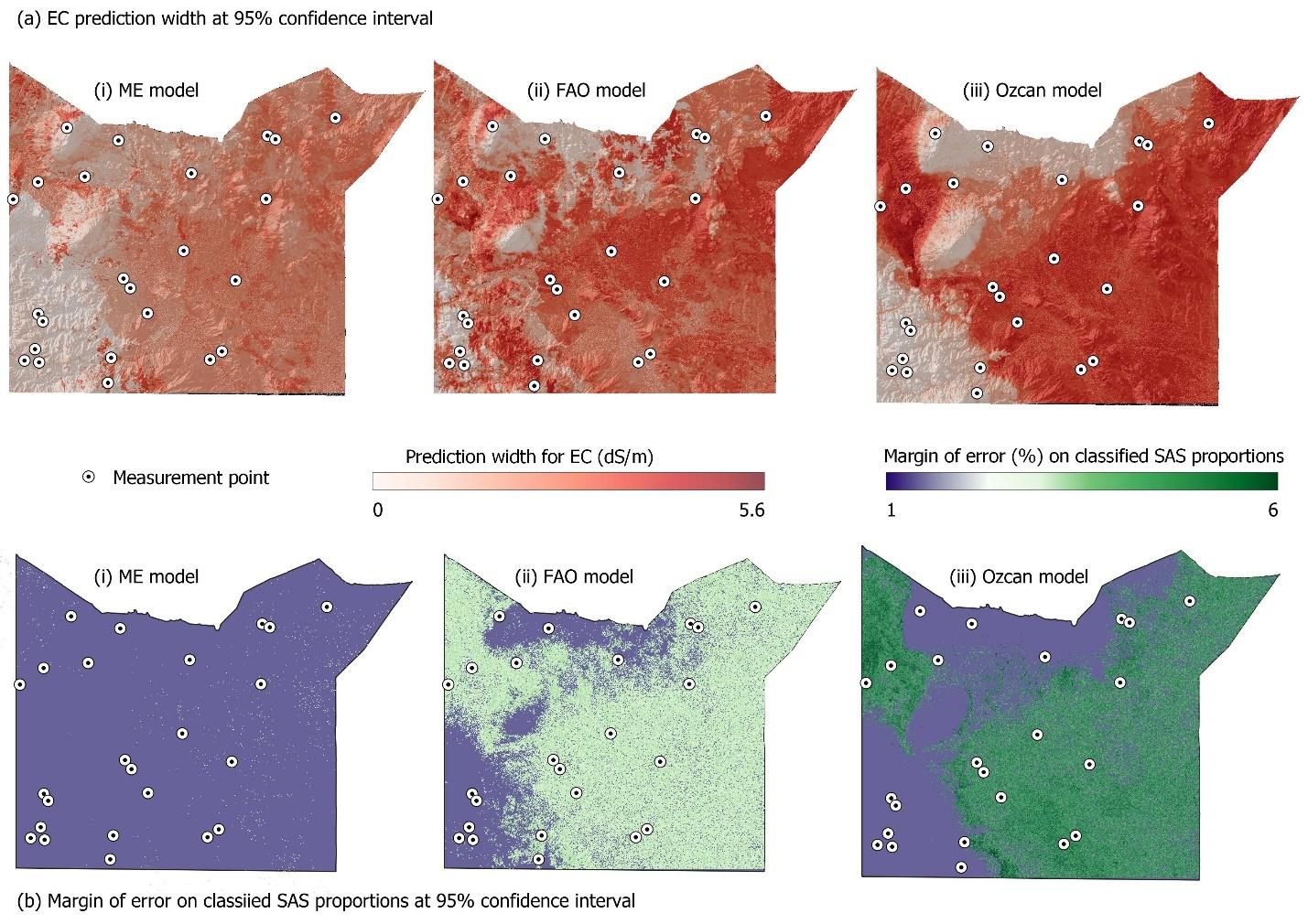


Figure S6.3: Example of uncertainty assessment for SAS information for north-east Kenya

Table S6.4: Areas of topsoil (0-30 cm) SAS in million hectares

| Region | Country | Moderate salinity | Strong salinity | Very strong salinity | Extreme salinity | Saline sodic | Slight sodicity | Moderate sodicity | Strong sodicity | Salinization risk areas | Total SAS |
| --- | --- | --- | --- | --- | --- | --- | --- | --- | --- | --- | --- |
| LAC | Bolivia | 0.23 | 0.11 | 0.03 | 0.01 | 0.01 | 0.04 | 0.01 |  | 1.96 | 0.44 |
|  | Peru | 1.83 | 1.83 | 2.53 | 0.63 |  |  |  |  | 2.02 | 6.82 |
|  | Ecuador | 1.76 | 0.18 | 0.02 |  | 0.27 | 0.96 | 0.03 |  | 0.74 | 3.22 |
|  | Jamaica |  |  |  |  |  |  |  |  | 0.07 | 0 |
|  | Trinidad |  |  |  |  |  |  |  |  | 0.003 | 0 |
|  | Cuba | 0.7 | 0.41 | 0.26 | 0.11 |  | 0.03 |  |  | 1.35 | 1.51 |
| Europe | Italy | 0.27 | 0.01 |  |  |  | 0.04 |  |  | 13.63 | 0.32 |
|  | Germany |  |  |  |  |  |  |  |  | 0.005 | 0 |
|  | Kazakhstan | 92.52 | 56.97 | 11.31 | 1.9 | 0.43 | 0.65 |  |  | 73.74 | 163.78 |
| Africa | Senegal | 2.47 | 1.75 | 0.03 |  |  |  |  |  | 1.71 | 4.25 |
|  | Nigeria | 0.85 |  |  |  |  |  |  |  | 6.4 | 0.85 |
|  | Kenya | 1.74 | 1.77 | 0.7 | 0.04 | 0.99 | 0.35 | 0.01 |  | 1.97 | 5.6 |
|  | South Africa | 4.47 | 0.51 | 0.58 | 0.02 | 0.06 | 0.01 | 0.01 | 0.05 | 17.7 | 5.71 |
|  | Rwanda | 0.01 |  |  |  |  |  |  |  | 0.13 | 0.01 |
| NENA | Oman | 10.62 | 5.03 | 0.14 |  | 0.01 | 0.02 |  |  | 4.46 | 15.82 |
|  | Jordan | 2.91 | 0.41 | 0.01 | 0.02 | 0.13 | 0.63 | 0.13 | 0.01 | 0.77 | 4.25 |
| Asia | Afghanistan | 5.6 | 0.61 | 0.11 | 0.02 | 0.17 | 0.12 |  |  | 4.86 | 6.63 |
|  | Pakistan | 13.82 | 1.31 | 0.02 |  |  |  |  |  | 15.42 | 15.15 |
|  | India | 22.74 | 3 | 0.07 | 0.01 | 0.19 | 0.24 |  |  | 86.14 | 26.25 |
|  | Bangladesh | 0.91 | 0.12 | 0.01 |  | 0.08 | 0.02 |  |  | 2.9 | 1.14 |
|  | Myanmar |  |  |  |  | 0 | 0.85 | 0.02 |  | 0 | 0.87 |
|  | Thailand | 0.27 | 0.02 |  |  | 0.45 | 3.43 | 0.76 |  | 0.79 | 4.93 |
|  | Cambodia | |  |  |  |  |  |  |  | 0.15 | 0 |
|  | Philippines | |  |  |  |  | 0.06 |  |  | 0.04 | 0.06 |
| Pacific | PNG |  |  |  |  |  | 0.02 |  |  | 0.08 | 0.02 |
|  | Samoa |  |  |  |  |  |  |  |  | 0.001 | 0 |

^*^ Areas classified as slight salinity (0.75≤ECse≤2 dS/m) by FAO^22^

#### References

1. Batjes, N. H., Ribeiro, E. & van Oostrum, A. Standardised soil profile data to support global mapping and modelling (WoSIS snapshot 2019). *Earth Syst. Sci. Data* **12**, 299–320. https://doi.org/ 10.5194/essd-12-299-2020 (2020).

2. Orgiazzi, A., Ballabio, C., Panagos, P., Jones, A. & Fernández‐Ugalde, O. LUCAS Soil, the largest expandable soil dataset for Europe: a review. *Eur. J. Soil Sci.* **69**, 140–153. https://doi.org/ 10.1111/ejss.12499 (2018).

3. FAO/IIASA/ISRIC/ISS-CAS/JRC. *Harmonized World Soil Database (version 1.2)*. (FAO and IIASA, 2012).

4. Soil Science Division Staff. Chapter 3: Examination and Description of Soil Profiles. in *Soil Survey Manual* 120–130 (Government Printing Office, 2017).

5. Laird, N. M. & Ware, J. H. Random-effects models for longitudinal data. *Biometrics* **38**, 963. https://doi.org/ 10.2307/2529876 (1982).

6. Lindstrom, M. J. & Bates, D. M. Nonlinear mixed effects models for repeated measures data. *Biometrics* **46**, 673. https://doi.org/10.2307/2532087 (1990).

7. Pinheiro, J. C. & Bates, D. M. *Mixed-Effects Models in Sand S-PLUS*. (Springer New York, 2000). doi:10.1007/978-1-4419-0318-1.

8. Allassonnière, S. & Chevallier, J. A new class of stochastic EM algorithms. Escaping local maxima and handling intractable sampling. *Comput. Stat. Data Anal.* **159**, 107159. https://doi.org/ 10.1016/j.csda.2020.107159 (2021).

9. Comets, E., Lavenu, A. & Lavielle, M. Parameter estimation in nonlinear mixed effect models using saemix , an r implementation of the SAEM algorithm. *J. Stat. Softw.* **80**. https:/doi.org/ 10.18637/jss.v080.i03 (2017).

10. Delyon, B., Lavielle, M. & Moulines, E. Convergence of a stochastic approximation version of the EM algorithm. *Ann. Stat.* **27**. https://doi.org/ 10.1214/aos/1018031103 (1999).

11. Krause, P., Boyle, D. P. & Bäse, F. Comparison of different efficiency criteria for hydrological model assessment. *Adv. Geosci.* **5**, 89–97. https://doi.org/ 10.5194/adgeo-5-89-2005 (2005).

12. Park, H.-J. *et al.* Assessment of electrical conductivity of saturated soil paste from 1:5 soil-water extracts for reclaimed tideland soils in South-Western coastal area of Korea. *Korean Soc. Environ. Agric.* **38**, 69–75. https://doi.org/10.5338/KJEA.2019.38.2.11 (2019).

13. Ozcan, H., Ekinci, H., Yigini, Y. & Yuksel, O. Comparison of four soil salinity extraction methods. in *Soil Sustaining Life on Earth, Managing Soil and Technology* 697–703 (2006).

14. Chi, C.-M. & Wang, Z.-C. Characterizing Salt-affected soils of songnen plain using saturated paste and 1:5 soil-to-water extraction methods. *Arid Land Res. Manag.* **24**, 1–11. https://doi.org/ 10.1080/15324980903439362 (2010).

15. Visconti, F., de Paz, J. M. & Rubio, J. L. What information does the electrical conductivity of soil water extracts of 1 to 5 ratio (w/v) provide for soil salinity assessment of agricultural irrigated lands? *Geoderma* **154**, 387–397. https://doi.org/ 10.1016/j.geoderma.2009.11.012 (2010).

16. Klaustermeier, A. *et al.* Comparison of soil-to-water suspension ratios for determining electrical conductivity of oil-production-water-contaminated soils. *Can. J. Soil Sci.* **96**, 233–243. https://doi.org/ 10.1139/cjss-2015-0097 (2016).

17. Richards, L. A. *Diagnosis and Improvement of Saline and Alkali Soils.* (USDA, 1954).

18. He, Y. *et al.* Evaluation of 1:5 soil to water extract electrical conductivity methods. *Geoderma* **185–186**, 12–17. https:/doi.org/10.1016/j.geoderma.2012.03.022 (2012).

19. Kargas, G. *et al.* Soil salinity assessment using saturated paste and mass soil:water 1:1 and 1:5 ratios extracts. *Water* **10**, 1589. https://doi.org/10.3390/w10111589 (2018).

20. Khorsandi, F. & Yazdi, F. A. Estimation of saturated paste extracts’ electrical conductivity from 1:5 soil/water suspension and gypsum. *Commun. Soil Sci. Plant Anal.* **42**, 315–321. https://doi.org/ 10.1080/00103624.2011.538885 (2011).

21. Sonmez, S., Buyuktas, D., Okturen, F. & Citak, S. Assessment of different soil to water ratios (1:1, 1:2.5, 1:5) in soil salinity studies. *Geoderma* **144**, 361–369. https://doi.org/ 10.1016/j.geoderma.2007.12.005 (2008).

22. FAO. *Guidelines for soil description*. (Food and Agriculture Organization of the United Nations, 2006).

23. Hogg, T. J. & Henry, J. L. Comparison of 1:1 and 1:2 suspensions and extracts with the saturation extract in estimating salinity in Saskatchewan soils. *Can. J. Soil Sci.* **64**, 699–704. https:/doi.org/ 10.4141/cjss84-069 (1984).

24. Aboukila, E. & Abdelaty, E. Assessment of saturated soil paste salinity from 1:2.5 and 1:5 soil-water extracts for coarse textured soils. *Alex. Sci. Exch. J.* **38**, 722–732. https://doi.org/ 10.21608/asejaiqjsae.2017.4181 (2017).

25. *Booker tropical soil manual: a handbook for soil survey and agricultural land evaluation in the tropics and subtropics*. (Booker Agriculture International Ltd. ; Longman, 1984).

26. Zhang, H., Schroder, J. L., Pittman, J. J., Wang, J. J. & Payton, M. E. Soil salinity using saturated paste and 1:1 soil to water extracts. *Soil Sci. Soc. Am. J.* **69**, 1146–1151. https://doi.org/ 10.2136/sssaj2004.0267 (2005).

27. Miller, R. O. & Kissel, D. E. Comparison of soil pH methods on soils of North America. *Soil Sci. Soc. Am. J.* **74**, 310–316 (2010).

28. Davies, B. E. A Statistical Comparison of pH values of some english soils after measurement in both water and 0.01m calcium chloride. *Soil Sci. Soc. Am. J.* **35**, 551–552. https://doi.org/ 10.2136/sssaj1971.03615995003500040022x (1971).

29. Brennan, R. F. & Bolland, M. D. A. Relationship between ph measured in water and calcium chloride for soils of Southwestern Australia. *Commun. Soil Sci. Plant Anal.* **29**, 2683–2689. https://doi.org/ 10.1080/00103629809370143 (1998).

30. Kabała, C., Musztyfaga, E., Gałka, B., Łabuńska, D. & Mańczyńska, P. Conversion of soil ph 1:2.5 kcl and 1:2.5 h2o to 1:5 h2o: conclusions for soil management, environmental monitoring, and international soil databases. *Pol. J. Environ. Stud.* **25**, 647–653. https://doi.org/ 10.15244/pjoes/61549 (2016).

31. Sadovski, A. N. Study on pH in water and potassium chloride for Bulgarian soils. *EURASIAN J. SOIL Sci. EJSS* **8**, 11–16. https://doi.org/ 10.18393/ejss.477560 (2019).

32. Ahern, C. R., Baker, D. E. & Aitken, R. L. Models for relating pH measurements in water and calcium chloride for a wide range of pH, soil types and depths. *Plant Soil* **171**, 47–52. https://doi.org/ 10.1007/BF00009563 (1995).

33. Minasny, B., McBratney, A. B., Brough, D. M. & Jacquier, D. Models relating soil pH measurements in water and calcium chloride that incorporate electrolyte concentration. *Eur. J. Soil Sci.* **62**, 728–732. https://doi.org/ 10.1111/j.1365-2389.2011.01386.x (2011).

34. Omuto, C. T. soilassessment: Assessment Models for Agriculture Soil Conditions and Crop Suitability. (2020).

35. Omuto, C. T. *et al.* Digital soil assessment in support of a soil information system for monitoring salinization and sodification in agricultural areas. *Land Degrad. Dev.* **33**, 1204–1218. https://doi.org/ 10.1002/ldr.4211 (2022).

36. Fick, S. E. & Hijmans, R. J. WorldClim 2: new 1‐km spatial resolution climate surfaces for global land areas. *Int. J. Climatol.* **37**, 4302–4315. https://doi.org/ 10.1002/joc.5086 (2017).

37. Earth Resources Observation And Science (EROS) Center. Shuttle Radar Topography Mission (SRTM) 1 Arc-Second Global. (2017) doi:10.5066/F7PR7TFT.

38. Conrad, O. *et al.* System for Automated Geoscientific Analyses (SAGA) v. 2.1.4. *Geosci. Model Dev.* **8**, 1991–2007. https://doi.org/10.5194/gmd-8-1991-2015 (2015).

39. Vermote, Eric. MOD09A1 MODIS/Terra Surface Reflectance 8-Day L3 Global 500m SIN Grid V006. (2015) doi:10.5067/MODIS/MOD09A1.006.

40. Gorji, T., Yıldırım, A., Sertel, E. & Tanık, A. Remote sensing approaches and mapping methods for monitoring soil salinity under different climate regimes. *Int. J. Environ. Geoinformatics* **6**, 33–49. https://doi.org/ 10.30897/ijegeo.500452 (2019).

41. Bishop, T. F. A., McBratney, A. B. & Laslett, G. M. Modelling soil attribute depth functions with equal-area quadratic smoothing splines. *Geoderma* **91**, 27–45. https://doi.org/ 10.1016/S0016-7061(99)00003-8 (1999).

42. Wang, J. *et al.* Soil salinity mapping using machine learning algorithms with the sentinel-2 msi in arid areas, China. *Remote Sens.* **13**, 305. https://doi.org/ 10.3390/rs13020305 (2021).

43. Abrol, I. P., Yadav, J. S. P. & Massoud, F. I. *Salt-Affected Soils and their Management*. (FAO, 1988).

44. Rossiter, D. G., Zeng, R. & Zhang, G.-L. Accounting for taxonomic distance in accuracy assessment of soil class predictions. *Geoderma* **292**, 118–127. https:/doi.org/ 10.1016/j.geoderma.2017.01.012 (2017).

45. Thai, H.-T., Mentré, F., Holford, N. H. G., Veyrat-Follet, C. & Comets, E. Evaluation of bootstrap methods for estimating uncertainty of parameters in nonlinear mixed-effects models: a simulation study in population pharmacokinetics. *J. Pharmacokinet. Pharmacodyn.* **41**, 15–33. https://doi.org/ 10.1007/s10928-013-9343-z (2014).

46. Efron, B. Bootstrap Methods: Another Look at the Jackknife. *Ann. Stat.* **7**. https://doi.org/ 10.1214/aos/1176344552 (1979).

47. Crowder, S., Delker, C., Forrest, E. & Martin, N. Monte Carlo Methods for the Propagation of Uncertainties. in *Introduction to Statistics in Metrology* 153–180 (Springer International Publishing, 2020). doi:10.1007/978-3-030-53329-8_8.
